# Genome-wide identification and analyses of the *AHL* gene family in cotton *(Gossypium)*

**DOI:** 10.1186/s12864-019-6406-6

**Published:** 2020-01-22

**Authors:** Lanjie Zhao, Youjun Lü, Wei Chen, Jinbo Yao, Yan Li, Qiulin Li, Jingwen Pan, Shengtao Fang, Jie Sun, Yongshan Zhang

**Affiliations:** 1 State Key Laboratory of Cotton Biology, Institute of Cotton Research of CAAS, Anyang, 455000 Henan China; 2 Research Base, Anyang Institute of Technology, State Key Laboratory of Cotton Biology, Anyang, 455000 Henan China; 3 Shihezi University, Shihezi, 832003 Xinjiang China

**Keywords:** Cotton, *AHL* family, AT-hook motif, Phylogenetics, Synteny, Expression profile, Ka/Ks

## Abstract

**Background:**

Members of the AT-HOOK MOTIF CONTAINING NUCLEAR LOCALIZED (*AHL*) family are involved in various plant biological processes via protein-DNA and protein-protein interaction. However, no the systematic identification and analysis of *AHL* gene family have been reported in cotton.

**Results:**

To investigate the potential functions of *AHLs* in cotton, genome-wide identification, expressions and structure analysis of the *AHL* gene family were performed in this study. 48, 51 and 99 *AHL* genes were identified from the *G.raimondii, G.arboreum* and *G.hirsutum* genome, respectively. Phylogenetic analysis revealed that the *AHLs* in cotton evolved into 2 clades, Clade-A with 4–5 introns and Clade-B with intronless (excluding *AHL*20–2). Based on the composition of the AT-hook motif(s) and PPC/DUF 296 domain, *AHL* proteins were classified into three types (Type-I/−II/−III), with Type-I *AHLs* forming Clade-B, and the other two types together diversifying in Clade-A. The detection of synteny and collinearity showed that the *AHLs* expanded with the specific WGD in cotton, and the sequence structure of *AHL20–2* showed the tendency of increasing intron in three different *Gossypium spp*. The ratios of non-synonymous (Ka) and synonymous (Ks) substitution rates of orthologous gene pairs revealed that the *AHL* genes of *G.hirsutum* had undergone through various selection pressures, purifying selection mainly in A-subgenome and positive selection mainly in D-subgenome. Examination of their expression patterns showed most of *AHLs* of Clade-B expressed predominantly in stem, while those of Clade-A in ovules, suggesting that the *AHLs* within each clade shared similar expression patterns with each other. qRT-PCR analysis further confirmed that some *GhAHLs* higher expression in stems and ovules.

**Conclusion:**

In this study, 48, 51 and 99 AHL genes were identified from three cotton genomes respectively. *AHLs* in cotton were classified into two clades by phylogenetic relationship and three types based on the composition of motif and domain. The *AHLs* expanded with segmental duplication, not tandem duplication. The expression profiles of *GhAHLs* revealed abundant differences in expression levels in various tissues and at different stages of ovules development. Our study provided significant insights into the potential functions of *AHLs* in regulating the growth and development in cotton.

## Background

The *AT-Hook Motif Containing Nuclear* *Localized* (*AHL*) gene family, a conserved transcription factor, has been found in all sequenced plant species [1]. The *AHL* proteins contain one or two AT-hook(s) and the Plant and Prokaryote Conserved (PPC/DUF296) domain, altering chromatin structure and regulate gene expressions [1–3]. AT-hook motif contains conserved Arg-Gly-Arg motif(s) binding to AT-rich DNA region. PPC domains contain 120 amino acids, sharing the same secondary or tertiary structure with seven β-sheets partially surrounding a single α-helix in a wide range of organisms ranging from prokaryotes to higher plants. The hydrophobic region at the C-terminal is essential to nuclear localization and interaction with each other or themselves [1, 2]. The *AHL* gene family regulates plant growth and development by forming DNA-protein and protein-protein homo−/hetero-trimeric complex [3, 4].

Phylogenetic analyses showed that *AHL* gene in land plants emerged in embryophytes and further diverged into two monophyletic clades predating the divergence of mosses from the rest of the land plants [1, 3]. The protein sequences of the PPC domain share unique characteristics within each of the two AHL phylogenetic clades. AT-hook motifs can be divided into two types. Based on type and number of the AT-hook motif(s) and the PPC domain, the AHL proteins can be further classified into three types [3]. In angiosperms, Clade-A *AHL*s expanded into five subfamilies; while, the ones in Clade-B expanded into four subfamilies [1].

The high conservation in molecule organization and evolution suggests a vital function of the *AHL* gene family in regulation of plant growth and development. Several studies have shown that the *AHL* gene family plays an important role in regulating the elongation of hypocotyl. In *A. thaliana*, some members of *AHL* gene family, such as *AHL6*, *AHL15*, *AHL22*, *AHL29* and *AHL27*, inhibits redundantly the elongation of hypocotyl by repressing the genes associated with auxin signaling [4–7]. *AHL* gene is related to floral transition and reproductive development of plants [8–12]. Overexpression of *GIANT KILLER* (*GIK*), which encodes for an *AHL* protein, leads to severe reproductive defects and down-regulation of genes involved in patterning and differentiation of reproductive floral organs [13]. *AtAHL22* delays flowering by acting as a chromatin remodeling factor that modifies the architecture of chromatin region of the *FT* gene by modulating both histone H3 acetylation and methylation [14, 15]. The *AHL* gene family also shows the instrumental role in maintaining hormones homeostasis and regulation of defense response in plants [16–19]. Studies have shown that there are 20 *AHL* genes in the rice genome data, which have three different expression patterns [19, 20]. All of the *OsAHL* genes might be functionally expressed genes with 3 distinct expression patterns [20]. Among them, in rice plants during both seedling and panicle development stages, overexpression of *OsAHL1* enhanced multiple stress tolerances, this gene could greatly improve drought resistance of rice plants [19].

Some studies on *AHL* family genes in plants are mainly focused on the model plant, *A. thaliana* and rice. Recently, Genome-wide, expression profiling, and network analysis of AT-hook gene family in maize will help in the further understanding the role of the this gene family in these this cereal crops [21]. Cotton is one of the most important fiber crop and provides amounts of natural fiber used for textile industry worldwide. Overexpression of *GhAT1*, the only reported *AHL* in cotton so far, facilitate the specification of fiber cells by repressing the activity of the lipid transfer protein gene *FSltp4* [22]. The *AHL* gene family in cotton remains a mystery to be solved. The completion of genome sequencing of cotton allows us for comprehensive identification and analysis of gene family in cotton [23–27]. Here, *AHLs* gene family from three cotton species genome datas were identified by bioinformatics methods, the gene structure features, chromosomal locations, phylogenetic relationships, synteny and expression profiles were illustrated to highlight the potential functional diversity. This study will enhance our understanding of the *AHL* gene family and providing insight into the potential functional diversity of *AHL* genes of *Gossypium.*

## Results

### Identification and features of *AHL* genes in cotton

To identify the *AHL* genes, the Blastp and Hmmer search program (HMMER3.0 package) was performed against the protein databases using the AtAHL protein sequences as queries. The candidate *AHL* genes were confirmed using PROSITE and InterProscan 65.0 software to search for the PPC and the AT-hook motifs. Finally, 12, 15, 21, 48, 51, 99 *AHL* genes were obtained from *Physcomitrella patens* (*P. patens*), *Vitis vinifera* (*V. vinifera*), *Theobroma cacao* (*T. cacao*), *Gossypium raimondii* (*G. raimondii*), *Gossypium arboreum* (*G. arboreum*), *Gossypium hirsutum* (*G. hirsutum*), respectively. The properties of identified *AHL*s in cotton were also analyzed by ExPASy (https://web.expasy.org/compute_pi/). The gene lengths of *AHL* genes in *G.raimondii* ranged from 684 bp to 8394 bp, which encoding polypeptides from 227 to 396 amino acid with predicted molecular weights ranging from 22.75 kD to 41.38 kDa. The theoretical pI ranged from 5.35 to 10.68 with charge from − 4 to 18 (Table 1). The *AHL* genes in *G.arboreum* and *G.hirsutum* differed greatly in length (641–10,972 bp), isoelectric point (5.3–10.6), molecular weight (17.22–45.29 kDa) and charge (− 5–19) (Additional files 1, 2).

**Table 1:** Information of the *AHL* genes in *G. raimondii*

Gene name	Sequence ID	Gene (bp)	CDS (bp)	Protein(aa)	Intron	MW^a^ (kDa)	pI^b^	Charge
*GrAHL1–1*	Gorai.003G167100.1	2846	984	327	4	33.734	9.912	8.5
*GrAHL1–2*	Gorai.004G203700.1	4309	1026	341	4	35.305	9.994	8.5
*GrAHL1–3*	Gorai.007G021700.1	3420	984	327	4	33.742	10.038	9.5
*GrAHL3*	Gorai.008G283600.1	3507	1008	335	4	35.308	8.45	6.5
*GrAHL5–1*	Gorai.004G211500.1	3767	1026	341	4	35.485	10.638	18
*GrAHL5–2*	Gorai.008G246700.1	4352	1023	340	4	35.256	10.307	16
*GrAHL7–1*	Gorai.004G161300.1	3148	1011	336	4	34.954	9.402	6
*GrAHL7–2*	Gorai.007G091400.1	4226	996	331	4	34.149	9.686	5.5
*GrAHL9–1*	Gorai.004G158000.1	3982	1023	340	4	35.007	9.669	10
*GrAHL9–2*	Gorai.008G122100.1	2887	990	329	4	34.067	10.453	14
*GrAHL9–3*	Gorai.007G098600.1	4773	1023	340	4	34.978	10.486	14
*GrAHL10*	Gorai.002G112700.1	6764	1095	364	4	37.03	10.27	14
*GrAHL13–1*	Gorai.004G186000.1	4606	1191	396	4	41.381	10.057	16
*GrAHL13–2*	Gorai.008G227100.1	3931	1176	391	4	40.457	9.13	8
*GrAHL14–1*	Gorai.007G280000.1	6475	1035	344	5	35.976	9.59	10.5
*GrAHL14–2*	Gorai.007G345200.1	4396	1038	345	5	36.075	9.86	12
*GrAHL14–3*	Gorai.013G186600.1	3669	1035	344	5	36.192	9.356	11.5
*GrAHL-X1*	Gorai.002G160000.1	5307	1101	366	4	38.034	9.785	9
*GrAHL-X2*	Gorai.006G158700.1	2517	1053	350	4	36.311	8.883	8.5
*GrAHL-X3*	Gorai.009G408800.1	3520	993	330	4	33.683	8.456	4
*GrAHL-X4*	Gorai.001G119100.1	2223	1095	364	4	38.243	7.851	4.5
*GrAHL-X5*	Gorai.012G024700.1	8394	633	210	4	22.749	5.77	−1.5
*GrAHL15*	Gorai.011G267800.1	3780	933	310	0	32.515	5.898	−4
*GrAHL16–1*	Gorai.006G007800.1	771	771	256	0	27.271	9.111	7.5
*GrAHL16–2*	Gorai.007G070000.1	1215	759	252	0	26.816	8.113	5
*GrAHL17–1*	Gorai.001G133900.1	1721	894	297	0	30.26	9.235	10.5
*GrAHL17–2*	Gorai.005G096700.1	921	921	306	0	31.725	7.472	5.5
*GrAHL17–3*	Gorai.006G120100.1	684	684	227	0	24.17	7.234	3
*GrAHL17–4*	Gorai.006G124100.1	684	684	227	0	24.232	6.944	2
*GrAHL17–5*	Gorai.009G075100.1	1020	882	293	0	30.273	8.108	8.5
*GrAHL17–6*	Gorai.009G230300.1	7250	957	318	0	33.38	8.214	11.5
*GrAHL17–7*	Gorai.010G035300.1	1448	864	287	0	29.728	7.054	3
*GrAHL17–8*	Gorai.013G253800.1	1304	987	328	0	33.21	10.682	10
*GrAHL20–1*	Gorai.005G048000.1	1403	888	295	0	30.487	5.349	−3.5
*GrAHL20–2*	Gorai.006G247900.1	1008	852	283	0	28.921	5.62	−3
*GrAHL20–3*	Gorai.007G280400.1	909	909	302	0	30.368	5.954	−2
*GrAHL22–1*	Gorai.001G173500.1	870	870	289	0	30.852	7.738	4
*GrAHL22–2*	Gorai.004G160700.1	1004	927	308	0	32.41	6.97	2
*GrAHL22–3*	Gorai.007G091800.1	1768	903	300	0	31.472	6.512	0
*GrAHL23–1*	Gorai.003G181200.1	804	804	267	0	27.75	6.856	2
*GrAHL23–4*	Gorai.008G226900.1	1477	801	266	0	27.957	6.739	1.5
*GrAHL23–2*	Gorai.004G185900.1	960	864	287	0	29.853	6.794	1.5
*GrAHL23–3*	Gorai.006G216300.1	828	828	275	0	28.779	6.221	−2
*GrAHL24–1*	Gorai.003G167700.1	1374	930	309	0	33.106	6.798	2.5
*GrAHL24–2*	Gorai.006G211500.1	900	900	299	0	31.775	6.704	1.5
*GrAHL24–3*	Gorai.008G240700.1	1751	924	307	0	32.791	6.584	0.5
*GrAHL25–1*	Gorai.005G215400.1	1776	846	281	0	28.239	9.008	4.5
*GrAHL25–2*	Gorai.012G138000.1	1800	846	281	0	28.419	8.982	5

^a^Molecular weight of the amino acid sequence, ^b^Isoelectric point

### Phylogenetic analysis and gene structures of *AHL* genes

**Fig. 1:**
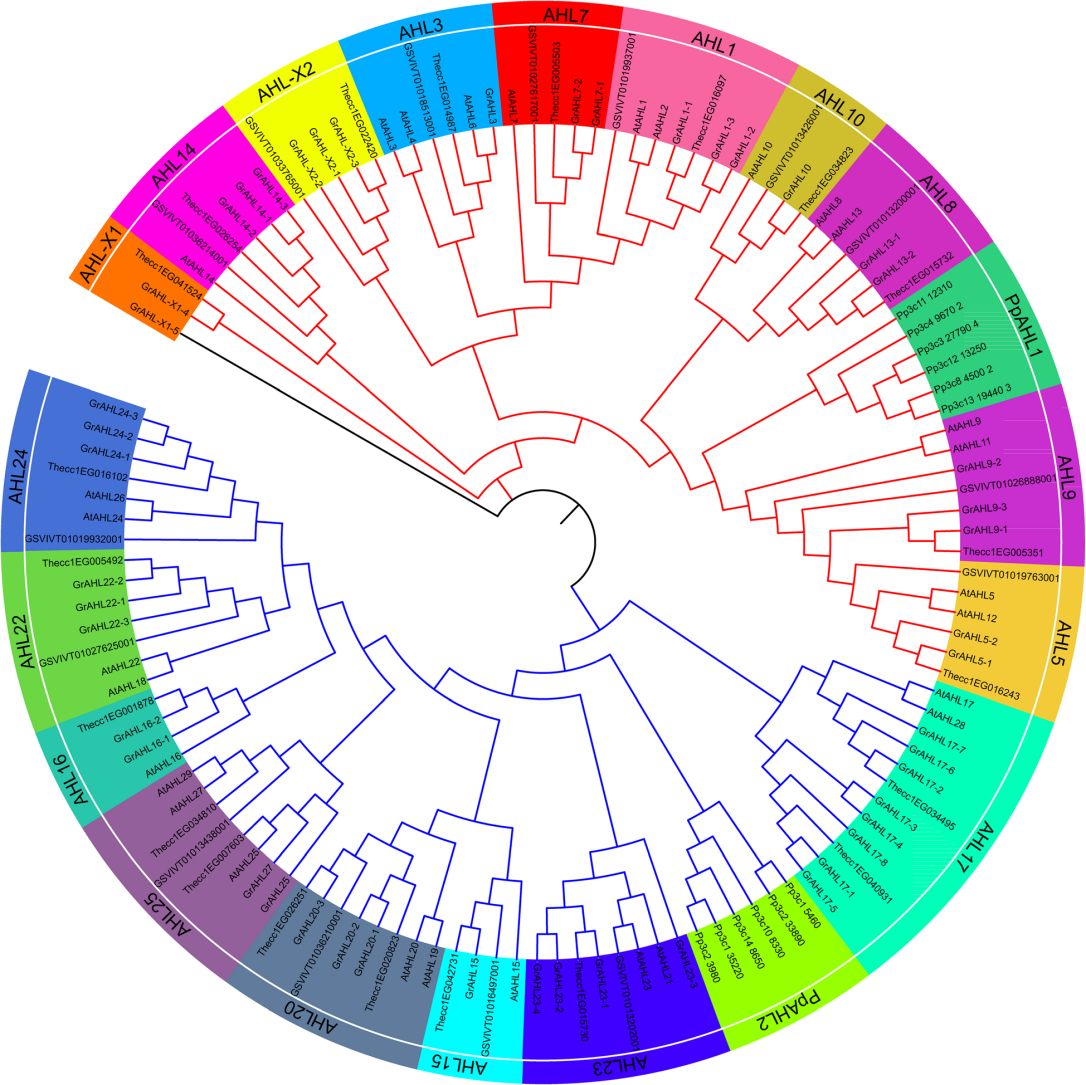
Phylogenetic relationship among *AHL* proteins. The maximum-likelihood (ML) phylogenetic tree was constructed by MEGA 7.0 using 1000 bootstrap replicates for the AHL proteins from *V. vinifera (Vv), A. thaliana (At), T. cacao (Tc), G. raimondii (Gr)* and *P. patens (Pp)*. Clade-A indicated in blue branch lines and Clade-B in red branch lines. The black line showed the pesudogene

To elucidate the evolutionary relationship of the *AHL* gene family in *Gossypium*, the maximum-likelihood phylogenetic tree was reconstructed by 1000 bootstrap replicates with *AHL* proteins from *P. patens* (*Pp*), *A. thaliana* (*At*)*, V. vinifera* (*Vv*), *T. cacao* (*Tc*) and *G. raimondii* (*Gr*). The phylogenetic analysis showed that the *AHL*s were divided into two monophyletic clades, Clade-A and Clade-B, with 9 and 8 groups respectively (Fig. 1). Each group in Clade-A (except for AHL-X*,* no corresponding *AHL* gene in *A. thaliana,* named AHL-X) was composed of one *VvAHL*, one *TcAHL*, different number of *AtAHL* and *GrAHL* respectively. Those groups in Clade-B were composed of various number of *AHL* genes from *A. thaliana (At), V. vinifera (Vv)*, *T. cacao (Tc)* and *G. raimondii (Gr)*. In *G .raimondii*, Clade-A contained 22 genes including the members of *GrAHL1, GrAHL3, GrAHL5, GrAHL7, GrAHL9, GrAHL10, GrAHL13, GrAHL14, GrAHL-X1 and GrAHL-X2,* while Clade-B contained 26 members including *GrAHL15, GrAHL16, GrAHL17, GrAHL20, GrAHL22, GrAHL23, GrAHL24* and *GrAHL25*. Each group of *GrAHL* gene family usually contained 2–3 members, while the group of *GrAHL17* had 8 members. The Group *AHL15, AHL10* and *AHL3* showed a more rigorous evolution pattern, with only one copy left in the genomes of the 4 examined species (Fig. 1, Additional file 3). This result indicated the different characteristics and the patterns of evolution in various group. The members of *AHLs* in *G. raimondii*, *G.arboreum* and *G.hirsutum* showed the preferably relationship of one-to-one correspondence except for *AHL17* and *AHL23*, there were 4 AHL17 members in *G. raimondii*, 6 in *G. arboreum*, and 9 in *G. hirsutum* (Additional file 4). The *AHLs* from *P. patens* evolved into two clades, suggesting an expansion of the *AHL* gene family in land plants posterior to the division between *P. patens* and the extant land plants [3].

### Conservation of gene structure and motifs among AHLs in cotton

**Fig. 2:**
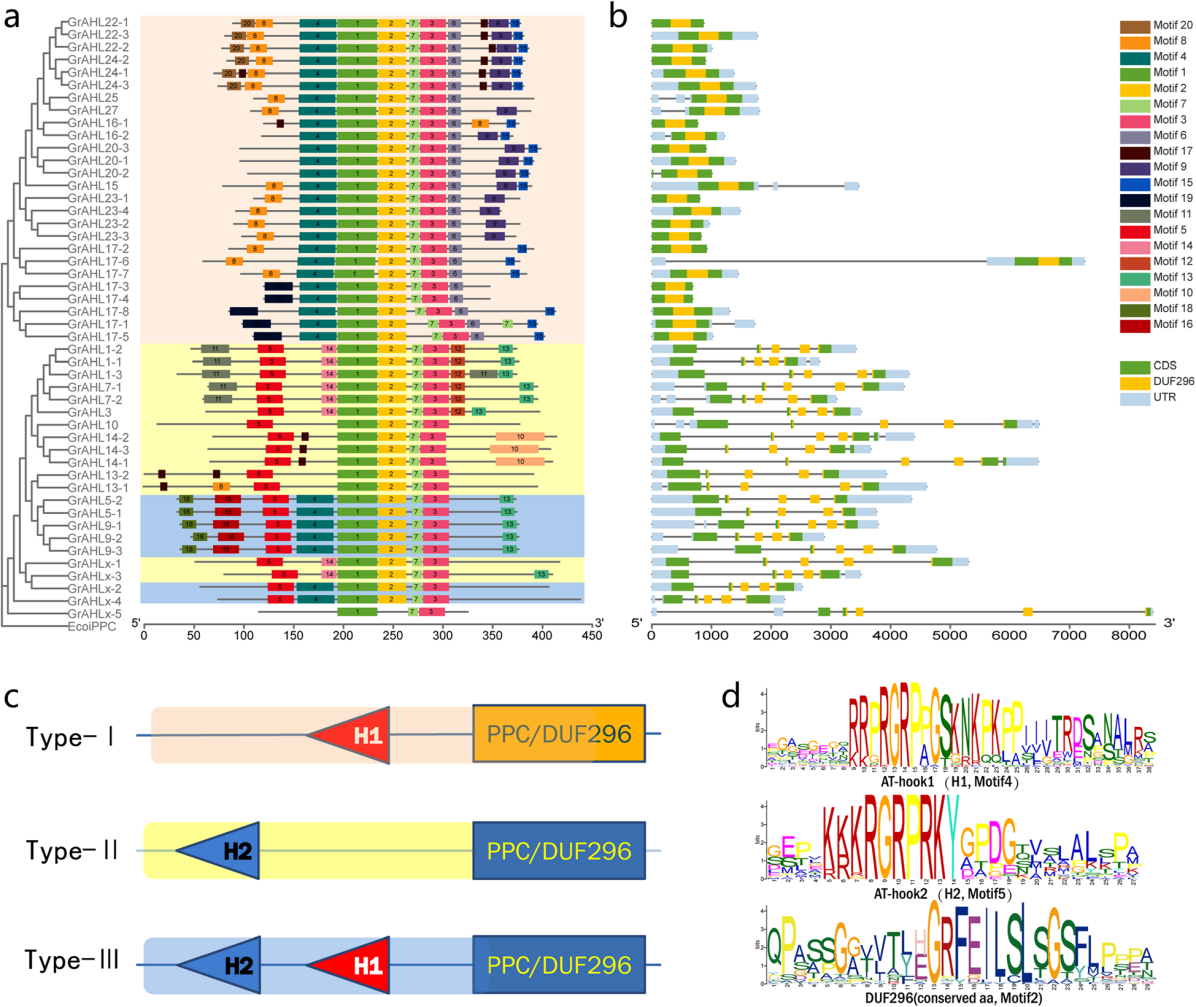
The conserved motifs, Exon–intron structures of *GrAHLs*. **a** The maximum-likelihood phylogenetic tree was constructed by MEGA 7.0 using 1000 bootstrap replicates. Conserved motifs and gene structure were predicted from MEME and GSDS website (http://meme-suite.org/tools/meme, http://gsds.cbi.pku.edu.cn/chinese.php). The length of proteins and DNA sequence was estimated using the scale at the bottom. The motifs were displayed in the different colored boxes with various numbers, black line indicated the non-conserved amino acid or introns. **b** Light blue boxes indicate untranslated 5- and 3-regions; green boxes indicate exons. **c** The PPC domains were highlighted by yellow boxes. The topology of three types of AHL proteins in cotton based on the AT-hook motifs and PPC domain (motif 2). H1 represented the AT-hook1 containing the conserved peptide in motif4; H2 represented the AT-hook2 containing the conserved peptide in motif5. **d** The conserved motifs  analysis of sequence logo

The AHL proteins were typically characterized by the presence of AT-hook motif(s) for binding DNA and PPC/DUF296 domain for nuclear localization and interaction with each other or themselves [4]. To investigate the presence of homologous domain sequences and the degree of conservation in the two domains, AT-hook motif(s) and PPC domain, we performed multiple sequence alignment to generate sequence logos of the two domains in cotton against the MEME website (http://meme-suite.org/tools/meme). 20 conserved motifs were predicted, and the specific amino acid sequences of each motif were also provided (Fig. 2, Additional file 8). Based on the number and composition of the AT-hook motif(s) and PPC/DUF 296 domain, AHL proteins were classified into three types (Type-I/−II/−III), with Type-I AHLs forming Clade-B, and the other two types together diversifying in Clade-A. Two types of AT-hook motifs (H1 and H2) were found in the AHL proteins (Fig. 2). Both of H1 and H2 in the AHL proteins shared the same conserved R-G-R-P core, showing that the ability of bind the minor groove of AT-rich B-form DNA. The conservation of H2 with a longer core R-G-R-P-R-K-Y heptapeptides was higher than that of the H1 in cotton. H1 was found only in Clade-B, while H2 or H1 plus H2 were found in Clade-A (Fig. 2). The *AHL* proteins in *T. cacao*, the closest related species of cotton, contained three types, while the AHL proteins in grape has only two types, Type-I and -III. The conserved structure of *AHL9, AHL5* from 4 species contained 2 AT-hook motifs, indicating the distinct function in development. Almost all *AHL* genes in cotton (except for *Gorai.012G0247001, Ga04G1890.1, Gh_D04G0182.1 and Gh_A05G3407.1,* named *AHL-X5*) contained AT-hooking motif (s) and PPC/DUF296 domain. We considered *AHL-X5*s (Table 1, Additional files 1 and 2) in cotton as pseudogenes, because they contained the most regions of PPC/DUF296 domains although lacking the AT-hooking motifs and core sequences (motif 2), so these four genes were used as the members of *AHL* family for further analysis.

To investigate the diversity of gene structure, we performed multiple sequence alignment to generate the exons/intron pattern using the GSDS (http://gsds.cbi.pku.edu.cn/chinese.php). The structures of *AHL* genes can be divided into two types, with intronless and multiple-exon. The 26 *AHL* genes in Clade-B showed intronless in *G.raimondii*, while those in Clade-A with 5–6 exons (Fig. 2). Most of the *AHL* genes in both *G. arboreum* and *G. hirsutum* presented similar exon/intron gene structure. The exception was *AHL20–2* in Clade-B, which had only one exon in *G. raimondii*, but its orthologous genes in both *G. arboreum* and *G. hirsutum* showed 4–5 introns in CDS, this indicating that the rapid evolution with intron-insertion (Additional file 5).

### Chromosomal location and synteny analysis of AHL genes

**Fig. 3:**
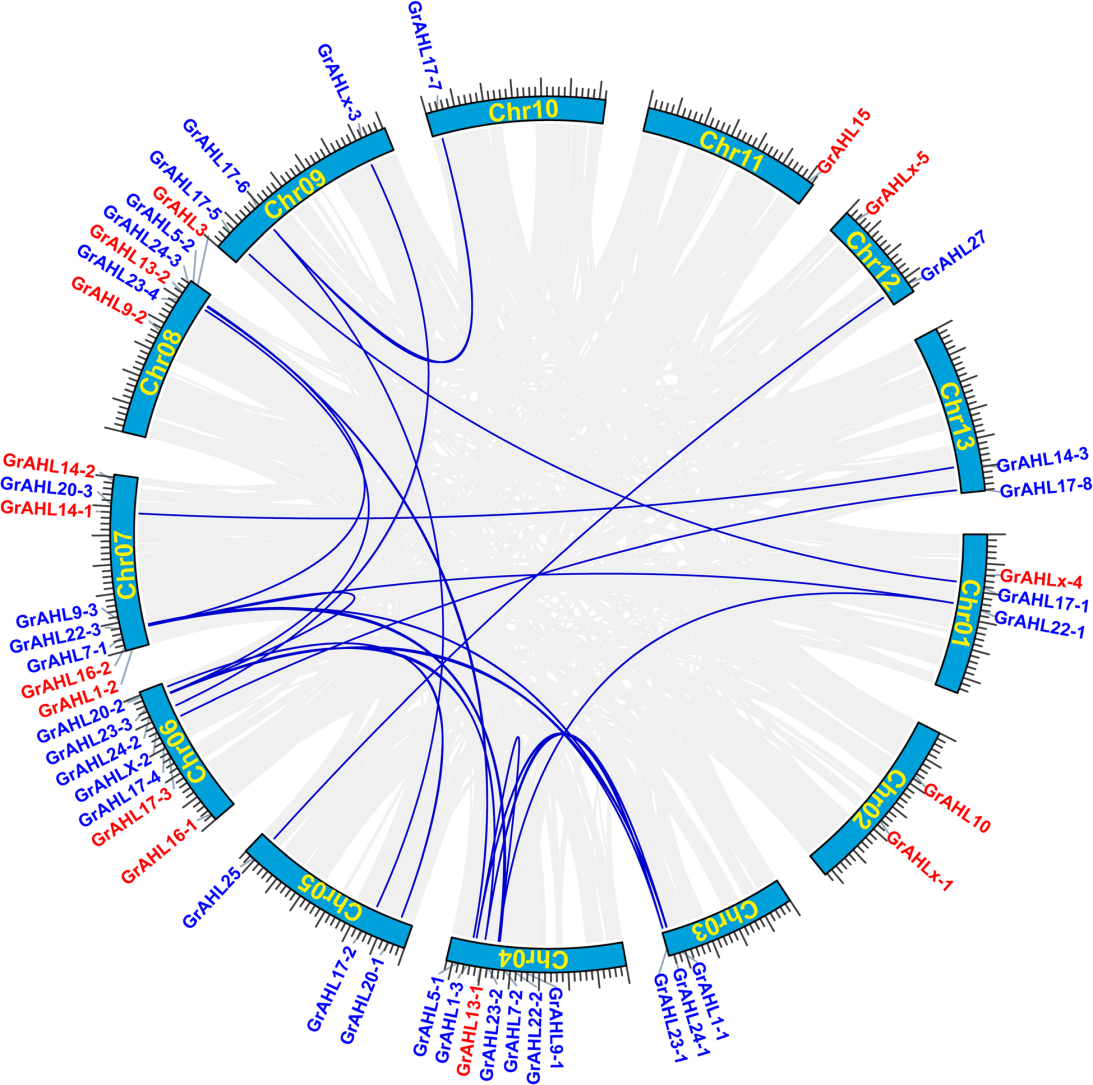
The synteny and collinearity analysis of *AHL* genes among grape, cacao, and cotton. The positions of AHL genes were depicted in chromosome of *V. vinifera* (*Vv*, orange band), *T. cacao* (*Tc*, blue band) and *G. raimondii* (*Gr*, red band). The Arabic numerals in bars represented different chromosome respectively. The picture was drawn with Circos

A total of 48 *GrAHL* genes were unevenly mapped onto 13 chromosomes of *G. raimondii*. Each chromosome contained 3–6 *AHL* members usually. Chromosome 07 contained 8 *AHL* genes, while chromosome 10 and 11 had only one *AHL*, respectively (Fig. 3). The distribution of *GaAHL* and *GhAHL* genes showed similar to *G. raimondii* but some *AHL* genes in scaffolds (Additional files 1 and 2).

**Fig. 4:**
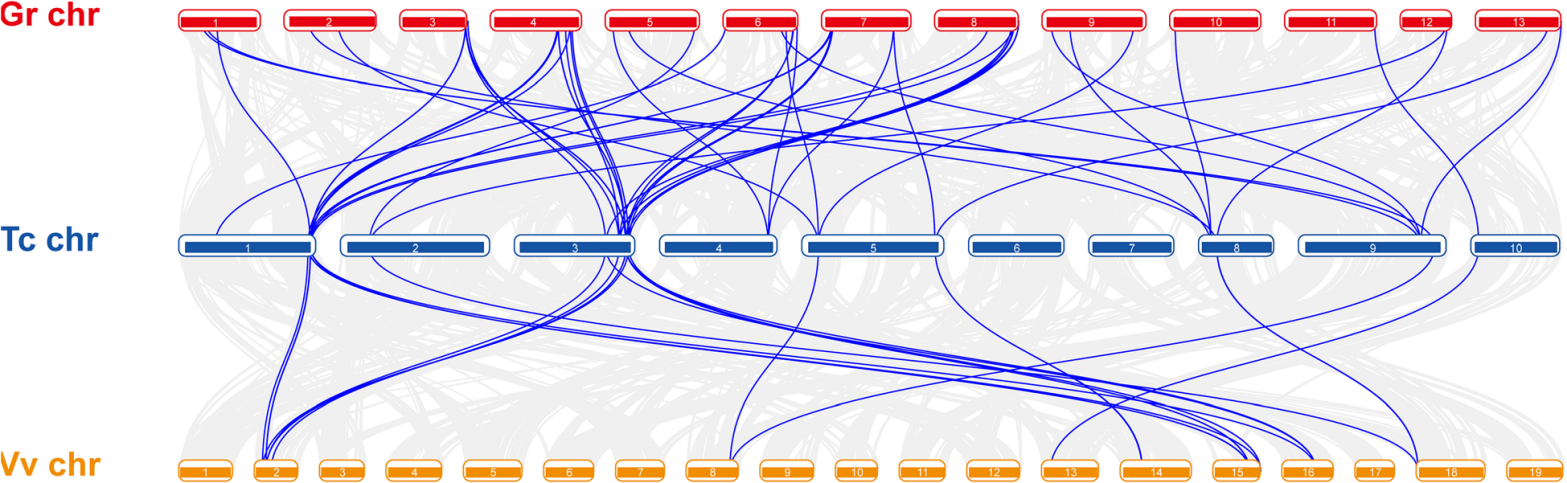
Distribution and gene duplications of *GrAHL* genes. The scale on the circle is in Megabases. Each colored bar represents a chromosome as indicated. Gene IDs are labeled on the basis of their positions on the chromosomes. *AHL* name in red indicated the singleton; AHL name in blue indicated the synteny or collinearity among chromosomes. The WGD or segmental duplication was linked by blue lines, gray lines in the background indicated the collinear blocks among different chromosomes

We surveyed the collinear relationship among the orthologous *AHL* genes from *V. vinifera*, *T. cacao* and *G. raimondii* to investigate the putative clues of evolutionary events. There were 15, 21, 29 and 48 *AHL* genes in *V. vinifera, T. cacao, A. thaliana* and *G. raimondii,* respectively. Specific loss and expansion of *AHL* genes were found in four species*. AHL16* and *AHL17* were not found in *V. vinifera,* while *AHL-X* not in *A. thaliana.* Most of *AHL* genes showed one-to-one corresponding relationship in *V. vinifera and T. cacao,* while 2–4 orthologous genes were found in *G. raimondii* (Fig. 3, Additional file 3). In order to investigate the pattern of gene duplication, MCScanX was used to analyze *AHL* gene family in *G. raimondii, G. arboreum* and *G. hirsutum*, the *AHL* genes in *G. raimondii* and *G. arboreum* showed the correspondent relationship between those from D-subgenome, A-subgenome in *G.hirsutum* respectively (Additional file 7)*.* The result indicated that the expansion of *GrAHL* gene family were with segmental duplication or whole genome duplication (WGD), no tandem duplication were found (Fig. 4).

### Different evolution of *AHL* genes in a and D subgenomes of *G. hirsutum*

**Fig. 5:**
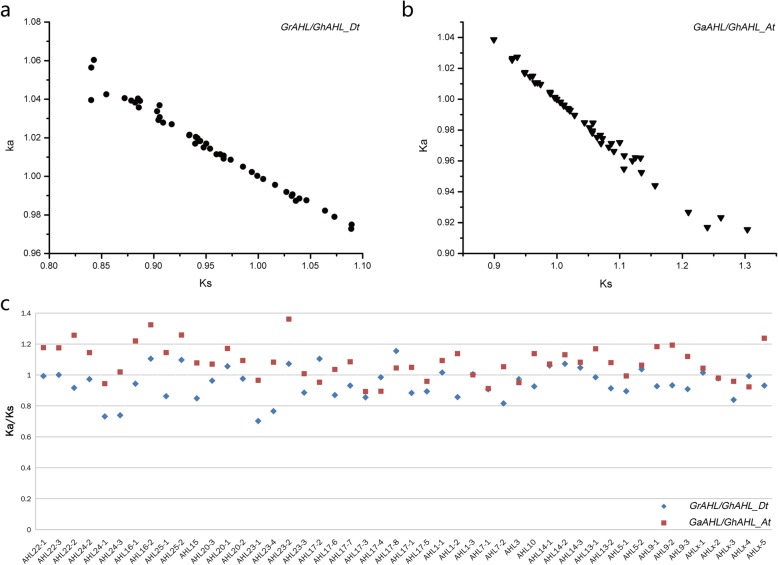
The distribution of non-synonymous (Ka) and synonymous (Ks) nucleotide substitution values of and Ka/Ks ratio of orthologous pairs between GrAHL, GaAHL and GhAHL. **a** Ks analysis of GrAHL/GhAHL_Dt. **b** Ks analysis of GaAHL/GhAHL_At. **c** Ka/Ks analysisof GrAHL/GhAHL_Dt and GaAHL/GhAHL_At

To explore the selective constrains among the orthologous *AHL* genes in *G. raimondii, G. arboreum* and *G. hirsutum*, we calculated Ks, Ka and the Ka/Ks ratio for the *AHL* gene pairs (Additional files 9 and 10). It is generally believed that the value of Ks was not affected by natural selection, but that of Ka was affected by natural selection. The Ka/Ks value can also explain positive selection (Ka/Ks > 1), neutral selection (Ka/Ks = 1) and negative selection (Ka/Ks < 1) during the evolution. In this study, 48 and 51 orthologous *AHL* gene paris were identified by OrthoMCL between *G. raimondii* and D-subgenome of *G. hirsutum* (*GrAHL/GhAHL_Dt*)*, and* bewteen *G. arboretum* and A-subgenome of *G. hirsutum* (*GaAHL/GhAHL_At*)*,* resprectively. The distributions of Ka and Ks between each pairs were shown in Fig. 5. The Ka of *GrAHL/GhAHL_Dt* ranged from 0.972745 to 1.08213, while Ks from 0.795064 to 1.08921. The Ka of *GaAHL/GhAHL_At* ranged from 0.915553 to 1.03866, while Ks from 0.899268 to 1.30387. 19 gene pairs of *GrAHL/GhAHL-Dt* (*39.6%*) with Ka/Ks > 1 were subjected to positive selection, while 2 (*AHL24–2* and *AHL17–3*) negative selection; 17 gene pairs of *GaAHL/GhAHL_At* (33.3%) with Ka/Ks < 1 were subjected to negative selection, while only *AHL17–8* positive selection. The result suggested that the *GhAHL* genes derived from *G. raimondii* and *G. arboreum* underwent various selection directions during the evolution.

### Gene expression profiles of *GhAHLs*

**Fig. 6:**
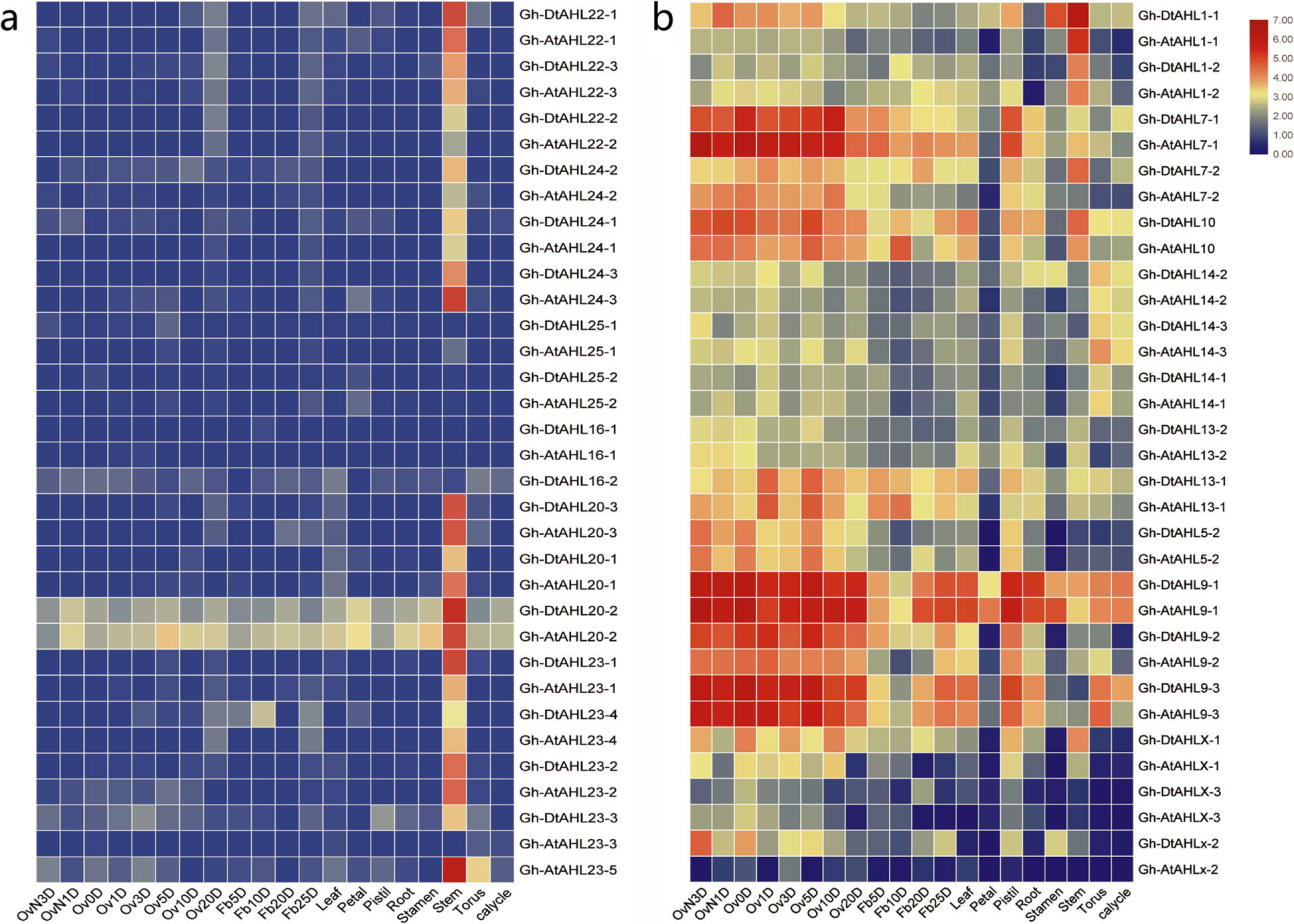
The expression profiles of *GhAHL* genes. The heatmap was generated on the basis of RNA-seq data from the website (http://www.cottonfgd.com), the color scale was shown at the right of the figure. Higher expression levels were shown in red, and lower in blue. OvN3D, represented the ovule in 3rd days before anthesis, Ov0d, represented the ovule in 0 day of anthesis, fb5d, = represented the fiber in 5th day after anthesis(**a**, **b**)

To explore the possible biological functions of *AHLs*, we inspected the expression patterns of different *AHL* genes in *G. hirsutum* based on the RNA-seq data downloaded from CottonFGD (http://www.cottonfgd.com). The *AHL* genes from *G. hirsutum* were expressed in different temporal and spatial patterns. Most *GhAHL* genes in Clade-B (such as *AHL20*, *AHL22*, *AHL23*, *AHL24)* were found strongly up-regulated expression in the stem, but extremely lowly in fiber, ovule, leaf, petal, root, stamen and pistil. Some of *GhAHL* genes in Clade-A (such as, *AHL1*, *AHL7*, *AHL9* and *AHL10)* showed an extensive expression activity in different organs, highly expressed in the fiber and ovule, suggesting a special function of these genes in the development of cotton ovule (Fig. 6). Interestingly, two *AHL20–2* genes inserted by introns in *G. hirsutum* showed higher expression activity in all organs and periods than other member in Clade-B. The expression of *GrAHL* showed similar pattern in different tissues (Additional file 6). The expression result showed that the *AHL*s within each clade shared similar expression patterns with each other; however, *AHL*s in one monophyletic clade exhibited distinct expression patterns from those in the other clade.

**Fig. 7:**
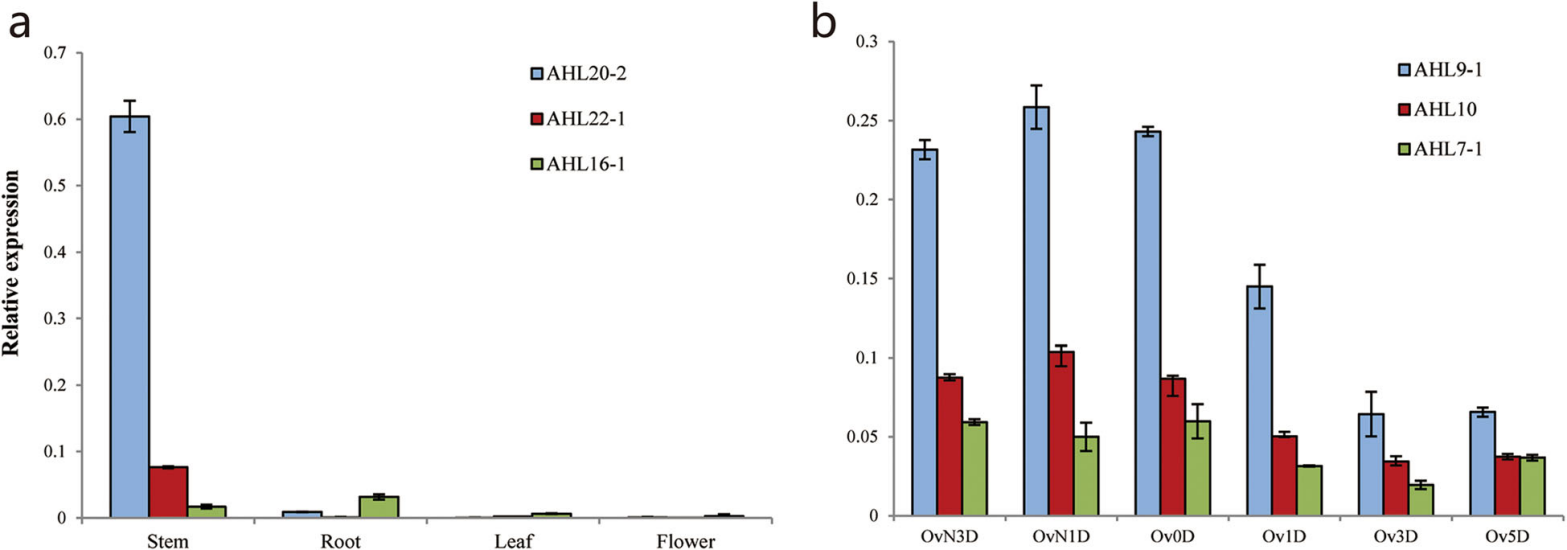
The expression patterns of six *AHL* genes in *G.hirsutum.* qRT-PCR was conducted to analyze the relative expression of six *AHL* genes in stem, root, leaf, flower and ovule (− 3,-1,0,1,3,5 DPA). **a** Expressed of *AHL22–1*, *AHL20–2* and *AHL16–1* genes in stem, root, leaf, flower. **b** Expressed of *AHL9–1*, *AHL7–1* and *AHL10* in − 3,-1,0,1,3,5DPA

For verification the data of RNA-seq, the qRT-PCR of six selected *AHL* genes in *G. hirsutum* was performed to analyze the expression pattern in stem, root, leaf, flower and ovule (− 3, − 1, 0, 1, 3, 5 DPA). The results showed that two *AHL* genes (*AHL22–1, AHL20–2*) in Clade-B displayed higher expression in stem, and lower expression in leaf. Three *AHL* genes (*AHL9–1, AHL7–1*and *AHL10*) in Clade-A expressed highly in the early development of ovule. *AHL16–1* expressed extremely lower in stem, root, leaf and flower (Fig. 7). The result coincided with the data of the RNA-seq, suggesting that the data from CottonFGD (http://www.cottonfgd.com) were reliable.

## Discussion

Cotton is one of the most important economical crops worldwide, providing more than 90% of the natural fiber for textile industry. Previous research about the *AHL* gene family has been performed in *A. thaliana*, *P. patens* and other monocot and dicot plants. In this study, we performed a comprehensive identification of *AHL* genes in *G. raimondii*, *G. arboreum*, and *G. hirsutum*, with an aim of understanding the important and diverse roles of this gene family in regulation of growth and development in plants.

### Identification of AHL proteins

In our study, 48, 51 and 99 *AHL* genes were identified from the *G. raimondii*, *G. arboreum* and *G. hirsutum* genomes, respectively. According to the phylogenetic analysis and gene structure, Ga07G1158.1 were regard as the member of the group of *AHL9*, but noted as *AHL1* in Version 2 of *G. arboretum;* Gh_D11G0864.1 should be regard as the member of *AHL22*, not AHL18 in notation. The group of *AHL-X5* (Gorai.012G0247001, Ga04G1890 Gh_D04G0182 and Gh_A05G3407) in 3 cotton species showed similar structure, containing the most regions of PPC/DUF296 domains, but the lack of the AT-hooking motifs, so we regarded these four genes as pseudogenes and the members of *AHL* family for further analysis. GSVIVT01013438001 in grape containing AT-hooking motif, but lack of part sequences of PPC/DUF296 domains, were also regard as the member of *AHL* gene family, different to the study of Zhao et al. [1]. 12 *AHL* genes were obtained from *P. patens,* which differ from the 10 *AHL* genes in the previous study maybe because of the annotation version of genome sequencing. Genomes of *G. hirsutum* are derived from hybridization between D-subgenome of *G. raimondii* and A-subgenome in *G. arboreum* [23–26]. The 47 of 48 *GrAHL* genes were located onto 13 chromosomes, showing one to one corresponding relationship with those of D-subgenome in *G. hirsutum*. No member of *GhAHL* was located onto the Chromsome 06 in D-subgenome, while *Gh_Sca005047G03* was located on scaffolds. Based on the synteny analysis, we speculated that *Gh_Sca005047G03* was likely located on Chromosome 06 of D-subgenome. *GaAHL* genes showed better linearity relationship to those in A-subgenome, it was speculated that *Gh_Sca009301G01*, *Ga14G0362*, *Ga14G0408* and *Ga14G1507* were likely located on *Gh_A11*, *GaChr09*, *GaChr06* and *GaChr02*, respectively (Additional file 8).

The *AHL* genes are divided into Clade-A and Clade-B, but the group members of Clade-A and Clade-B were respectively 5 and 4 in land plants [1], more than those from *P. patens*, suggesting that an significant expansion of the *AHL* gene family in land plants. 48 *GrAHL* genes in *G. raimondii* were more than those from other species reported in previous report or closely-related species, such as *T. cacao* (21) and grape (15) [1]. Each group in Clade-A (except for AHL-X*)* was composed of one *VvAHL*, one *TcAHL*, different number of *AtAHL* and *GrAHL*, respectively. Most groups had 2–3 members in the diploid cotton, while the *GrAHL17* had 8 members in *G. raimondii,* 9 in *G. arboreum and 18 G. hirsutum,* indicating a different expansion of the *AHL* gene family. The synteny results showed that the expansion of *AHL* family were with the WGD or segmental duplications, not tandem duplication. Related research suggested that the ancestor of *Gossypium* experienced a whole-genome duplication event after its divergence from *T. coco* ancestor [23, 24]. So, we speculated that the numbers of the *AHL*s in *G. raimondii* or *G. arboreum* were more than that in *V. vinifera* and *T. cacao* maybe due to the specific WGD event in *Gossypium* ancestor after the divergence of cotton from *T. cacao* [23, 25]. The *AHL* gene losses were also found in *A. thaliana*, group *AHL-X* included the corresponding *AHL* genes from *G. raimondii*, *V. vinifera* and *T. cacao*, no member were found in *A. thaliana*, suggesting that the different number of each group resulted from the various gene loss.

### Conservation of the *AHL* gene family

The *AHL* gene family is a plant-specific family with conserved structure of AT-hook and PPC/DUF domain. The members of *AHL* family present diversity not only in the sequence of AT-hook and PPC motifs, but also in gene length, gene structure, as well as in motif number. An analysis of sequence logo was performed for further investigating the divergence of AT-hook motif and the PPC domains in AHL proteins. AT-hook motif (s) could be distinguished by the phylogenetic relatedness of its homeodomains. Our results demonstrated that a longer core sequence R-G-R-P in *AHL* proteins in cotton, especially in type II AT-hook motif, containing a more longer and conserved core R-G-R-P-R-K-Y heptapeptide. According to the AT-hook motif and PPC domain, the *AHL* proteins in cotton were divided into three types, agree with previous study [1, 3]. Two types of gene structure, with intronless and multiple-exon, were found in the *AHL* genes of cotton. The 26 *GrAHL* genes in Clade-B showed intronless, while those genes in Clade-A with 5–6 exons. The *AHL* genes in *V. vinifera* presented another scenario, in which most of the *AHL* genes contain multiple exons except for the sole-exon gene *GSVIVT01027625*. There were some exceptions in cotton and *T. cacao*, such as the inclusion of multiple exons in *T. cacao Thecc1EG005492* and *Thecc1EG034810*, which were clustered in Clade-A. The difference of gene structure among the *AHL20–2* genes in different cotton species were showed in Fig. S4, *GrAHL20–2* possessed only one exon while its orthologous members in *G. arboreum* and *G. hirsutum* contained multiple introns, suggesting a rapid evolutionary rate during the history of cotton. Furthermore, the *AHL* genes in Clade-B in *G. hirsutum* were mainly specifically expressed in stem, with no detectable expression in other organs. Two members of *AHL20–2* from A-subgenome and D-subgenome respectively, with multiple introns, expressed in various organs and tissues, suggesting that the gene structure may have some effects on gene expression pattern.

### Expression profile analysis of AHL in cotton

The *AHL* genes play important roles in plant development, floral transition and response to biotic and abiotic stress [1, 4, 10]. *AHL20*, *AHL22*, *AHL23* and *AHL24* were strongly up-regulated expressed in the stem, but extremely lowly in fiber, ovule, leaf, petal, root, stamen and pistil. *AHL1*, *AHL7*, *AHL9* and *AHL10* showed an extensive expression activity in different organs, highly expressed in the fiber and ovule, suggesting a special function of these genes in the development of cotton ovule. According to the phylogenetic analysis, Group of AHL3, AHL10 and AHL15 kept one copy left in *V. vinifera*, *T. cacao*, *A. thaliana* and *G. raimondii*, suggesting the more conserved function or vital roles in development. The gene expression patterns of *GhAHL10* and *GhAHL15* were observably different, *GhAHL10* was observably expressed in all detected tissues and stages, while *GhAHL15* were not detected expression in any detected tissues and stages. Compared with the homologous groups of *V. vinifera* and *T. cacao*, the members of *AHL17* were observably expanded to 8 in *G. raimondii* and 17 in *G. hirsutum*; no expression was detected in tissues and stages except *AHL17–2* and *AHL17–6*, consistent with decreased gene expression levels after gene expansion in previous reports. The expression result showed that the *AHL*s within each clade shared similar expression patterns with each other; however, *AHL*s in one monophyletic clade exhibited distinct expression patterns from the ones in the other clade.

## Conclusions

Previous studies have shown that the *AHL* genes play important roles in plant growth and development, and response to biotic and abiotic stress. This study provides a comprehensive analysis of *AHL* gene family in the genomes of three cotton species. All of the genes showed one-to-one homology relationship among three different genomes or subgenomes in cotton. Phylogenetic andSynteny analysis indicated that *AHL*s in cotton were highly homologous to those in *V. vinifera and T. cacao*. *AHL* genes are highly conserved among cotton and other plant species. Sequence analysis showed that segmental duplications were the major driving forces of *AHL* family evolution, suggesting that *AHLs* expanded with specific WGD in cotton. It is consistent with the identification and analysis results of the whole gene family of *AHLs* in maize. The ratios of non-synonymous (Ka) and synonymous (Ks) substitution rates between orthologous gene pairs revealed that the *AHL* genes of *G.hirsutum* had undergone through various selections during evolution, purifying selection mainly in A-subgenome and positive selection mainly in D-subgenome. A further expression analysis using RNA-seq transcriptome and qRT-PCR indicated that most of *AHLs* of Clade-B expressed predominantly in stem, while those of Clade-A in ovules, suggesting that the *AHLs* within each clade shared similar expression patterns within each other, those genes might have experienced a functional divergence. Our study provided a reference for the further functional investigation of these selected candidate *AHL* proteins.

## Methods

### Identification of the *AHL* genes

To identify the *AHL* gene family in cotton, the genome sequence and annotation data of four cotton species, including *G. raimondii* [23, 24], *G. arboreum* [25], *G. hirsutum* [26] and *G. barbadense* [11], were obtained from the CottonFGD (http://www.cottonfgd.org/) [27] by blastp against protein database and tblastn against genome databases using the query sequences of the 29 AHL proteins in *A. thaliana* acquired from TAIR 15 (http://www.arabidopsis.org), the E-value cut-off was set at 1.0e-5 to ensure confidence. The *AHL* genes from *P. patens* (*Pp*), *A. thaliana* (*At*)*, V. vinifera* (*Vv*), *T. cacao* (*Tc*) were retrieved from the Phytozome database v12.1 (https://phytozome.jgi.doe.gov/pz/portal.html). Redundant sequences were detected and deleted by manual. The candidate sequences were submitted to PROSITE for PPC domain (PS51742), those sequences comprised of AT-hook motif (s) and PPC domain were confirmed as *AHL* genes for further analysis. Protein sequences of *AHL* were submitted to ExPASy (http://web.expasy.org/protparam/) to predict the molecular weights (MW) and theoretical isoelectric points (pI) and charge.

### Chromosomal location and collinearity analysis

The information of the *AHLs* loci on chromosome was obtained from annotation gff3 files. The Gene Structure Display Server (http://gsds.cbi.pku.edu.cn/) was used for gene structure analysis. Conserved protein motifs of the *AHLs* were predicted by the MEME program (http://meme-suite.org/tools/meme). The parameters of MEME were optimum width, 3–50; number of repetitions, any; maximum number of motifs, 20. A schematic diagram of gene structure was redrawn by Circos. The MCscanX program was used to identify *GrAHL* duplications as previous described by Wang et al. [28]. Total 37,505 proteins sequences were used by all-all BLAST with e-value< 1.0e-5. All genes were classified into various types of duplications, dispersed, singleton, WGD or segmental and tandem duplications. A schematic diagram of the putative WGD or segmental duplications of *GrAHL* was constructed using the Circos, and the *AHLs* with WGD or segmental duplications were linked by lines.

### Phylogenetic analysis and classification of *AHL* genes in cotton

For phylogenetic analysis, All AHL amino sequences from *P. patens* (*Pp*), *A. thaliana* (*At*)*, V. vinifera* (*Vv*), *T. cacao* (*Tc*) and three cotton species were aligned by ClustalX v1.83 with default parameters [29]. MEGA 7.0 was used to find best model and construct the Maximum likelihood (ML) tree with bootstrap test of 1000 replicates, the model of JTT + G was selected as the best model. Neighbor-Joining (NJ) phylogenetic trees were also generated in MEGA 7.0 to validate the ML phylogenetic trees [30].

### Calculation of Ka/Ks of *AHL* genes in cotton

The orthologs of the *AHL* genes in *G. raimondii, G. arboreum* and *G. hirsutum* were identified by OrthoMCL [31]. The orthologous gene pairs of *AHLs* were aligned by codons with Muscle in MEGA 7.0 software. Non-synonymous (Ka) and synonymous (Ks) substitution rates and Ka/Ks ratios of were determined by the model average (MA) and model (MS) in Kaks_Calculator 2.0 program [32], respectively.

### Expression profiles of *GhAHL* genes

For analyzing the expression profile of *GhAHL* and *GrAHL* genes in different tissues and development stages, the expression data of fragments-per-kilobase-per-million (FPKM) were retrieved from the genome-wide RNA-seq dataset in CottonFGD (http://www.cottonfgd.com/data) and CCnet website (http://structuralbiology.cau.edu.cn/gossypium), respectively. For each RNA-seq analysis, transcripts were assembled using Cufflinks software [33]. The heatmap charts were drawn according to gene expression values (FPKM).

### Quantitative RT-PCR (qRT-PCR) for *GhAHL* genes

The upland cotton (TM-1) seeds were germinated on a wet germinated disc for 3 days at 28 °C, and then transferred to a liquid culture medium. Total RNA was extracted from the seedlings. The leaves, root and stem were collected and were immediately frozen in liquid nitrogen for RNA extraction. Blossom in full bloom, and then take the first 3 days, 1 day, 0, 1 days after flowering, flowering after 3 days, 5 days after flowering ovule and flower liquid nitrogen treatment − 80 °C after preservation; Total RNA was extracted from the seedlings. cDNA was synthesized by using an EASYspin Plus Plant RNA Kit (Aidlab) with gDNA Eraser (Takara). The qRT-PCR reactions were conducted using a SYBR Green I Master mixture (Roche, Basel, Switzerland) according to the manufacturer’s protocol on a Light Cycler 480II system (Roche, Switzerland). Cotton ACTIN14 (GenBank accession number: AY305733) was used as an internal control in the PCR assays. The primers designed for qRT-PCR were showed in Additional file 11. The qRT-PCR was completed with three biological replicates, each comprising three technical replicates. The PCR conditions were as follows: 95 °C for 30 s; 40 cycles of 95 °C for 5 s, 60 °C for 1 min, and 72 °C for 10 s; 50 °C for 30 s. The relative gene expression levels were calculated based on the 2^−ΔΔ*C*T^ method [34].

## Supplementary information

**Additional file 1. **- Information of *AHLs* in *G. arboretum.* a Molecular weight of the amino acid sequence, b Isoelectric point

**Additional file 2. **- Information of *AHLs* in *G. hirsutum*. a Molecular weight of the amino acid sequence, b Isoelectric point

**Additional file 3. **- The orthologous relationship and type of AHL proteins in *V. vinifera, T. cacao, A.thaliana* and *G. raimondii.* The forms in pink indicated the Type-I *AHL* genes, those in yellow indicated the Type-III *AHL* genes and those in blue indicated the Type-II *AHL* genes. The lines repented the loss of orthologous gene

**Additional file 4. **- Phylogenetic relationship of AHL proteins in cotton. *AHL* proteins from *G. raimondii*, *G. arboreum* and *G. hirsutum* are marked with blue rhombus, green squares, and red rhombus squares, respectively

**Additional file 5. **- The variations of gene structures and motifs of *AHL20–2* from *G. raimondii*, *G. arboreum* and *G. hirsutum.* Gene structure and conserved motifs were predicted from the GSDS and MEME website. The length of proteins and DNA sequence was estimated using the scale at the bottom. The motifs were displayed in different colored boxes with Arabic numerals; black line indicated the non-conserved amino acid or intron. Gray boxes indicate untranslated 5- and 3-regions, blue boxes indicate exons. The sequences of motifs were listed in additional file 6

**Additional file 6.** - The sequences of motifs predicted by MEME (http://meme-suite.org/tools/meme)

**Additional file 7. **- The expression profiles of *GrAHLs*. The heatmap was generated on the basis of RNA-seq data from the website (http://structuralbiology.cau.edu.cn/gossypium), the color scale was shown at the right. Higher expression levels were shown in red, and lower in blue. DPA represented the day of ovule after anthesis

**Additional file 8. **- The circos map of *AHL*s in *G. raimondii, G. arbretum* and *G. hirsutum.*The collinearity of *AHL* genes between *G. raimondii and D-subgenome in G. hirsutum* were showed in orange lines, that between *G. arbretum* and the A-subgenome in *G. hirsutum in blue lines. AHL* genes located in scaffolds were showed in red lines, and the locations of scaffolds were putatived

**Additional file 9. **- Ka, Ks and Ka/Ks ratio between orthologous genes pairs from *G. raimondii and D-subgenome in G. hirsutum*

**Additional file 10. **- Ka, Ks and Ka/Ks ratio between orthologous gene pairs from *G. arbretum and A-subgenome of G. hirsutum*

**Additional file 11.** - The primers designed for qRT-PCR

## Data Availability

All another data generated or analyzed during this study are included in this published article and its Additional files.

## Abbreviations

AHL: AT-Hook Motif Containing Nuclear Localized
BLAST: Basic Local Alignment Search Tool
DPA: Days post anthesis
FPKM: Fragments per kilobase of transcript per million mapped fragments
*G. arboreum*: *Gossypium arboreum*
*G. hirsutum*: *Gossypium hirsutum*
G. raimondii: Gossypium raimondii
GIK: GIANT KILLER
Ka: Non-synonymous
Ks: Synonymous
ML: Maximum likelihood
MW: Molecular weight
NJ: Neighbour Joining
*P. patens*: *Physcomitrella patens*
pI: Isoelectric point
qRT-PCR: Quantitative real-time polymerase chain reaction
*T. cacao*: *Theobroma cacao*
*V. vinifera*: *Vitis vinifera*
WGD: Whole genome duplication
