# Supplementary material for: Genome-wide identification and analyses of the AHL gene family in cotton (Gossypium)
Source: BMC Genomics. 2020 Jan 22;21:69. doi: 10.1186/s12864-019-6406-6 (PMC6977275; doi:10.1186/s12864-019-6406-6)
Supplement: Supplementary file 1 — Additional file 1. - Information of AHLs in G. arboretum. a Molecular weight of the amino acid sequence, b Isoelectric point [file 12864_2019_6406_MOESM1_ESM.docx]

**Additional file 1-** Information of *AHLs* in G. *arboreum*

| Gene Name | Sequence ID | Gene (bp) | CDS (bp) | Protein (aa) | Intron | MW  (kDa) | pI | Charge |
| --- | --- | --- | --- | --- | --- | --- | --- | --- |
| *GaAHL22-1* | Ga07G1626.1 | 870 | 870 | 289 | 0 | 30.925 | 8.701 | 5.5 |
| *GaAHL22-3* | Ga11G3210.1 | 906 | 906 | 301 | 0 | 31.656 | 6.512 | 0 |
| *GaAHL22-2* | Ga08G1595.1 | 921 | 921 | 306 | 0 | 32.155 | 6.728 | 1 |
| *GaAHL24-2* | Ga09G2178.1 | 900 | 900 | 299 | 0 | 31.776 | 6.753 | 2 |
| *GaAHL24-1* | Ga01G2674.1 | 936 | 936 | 311 | 0 | 33.285 | 6.966 | 4 |
| *GaAHL24-3* | Ga12G0567.1 | 924 | 924 | 307 | 0 | 32.778 | 6.584 | 0.5 |
| *GaAHL25-1* | Ga03G2240.1 | 849 | 849 | 282 | 0 | 28.382 | 8.462 | 3.5 |
| *GaAHL25-2* | Ga04G0373.1 | 852 | 852 | 283 | 0 | 28.507 | 8.982 | 5 |
| *GaAHL16-1* | Ga09G0074.1 | 771 | 771 | 256 | 0 | 27.287 | 9.111 | 7.5 |
| *GaAHL16-2* | Ga11G3433.1 | 759 | 759 | 252 | 0 | 26.852 | 8.486 | 6 |
| *GaAHL15* | Ga10G0266.1 | 933 | 933 | 310 | 0 | 32.555 | 5.701 | -5 |
| *GaAHL20-3* | Ga11G1043.1 | 909 | 909 | 302 | 0 | 30.377 | 5.954 | -2 |
| *GaAHL20-1* | Ga03G0429.1 | 888 | 888 | 295 | 0 | 30.453 | 5.349 | -3.5 |
| *GaAHL20-2* | Ga09G2561.1 | 6,987 | 1,248 | 415 | 6 | 44.475 | 8.769 | 9 |
| *GaAHL23-1* | Ga01G2727.1 | 804 | 804 | 267 | 0 | 27.806 | 6.912 | 2.5 |
| *GaAHL23-4* | Ga12G0711.1 | 801 | 801 | 266 | 0 | 27.943 | 6.739 | 1.5 |
| *GaAHL23-2* | Ga08G1854.1 | 864 | 864 | 287 | 0 | 29.754 | 6.793 | 1.5 |
| *GaAHL23-3* | Ga09G2227.1 | 816 | 777 | 258 | 1 | 26.898 | 6.415 | -0.5 |
| *GaAHL23-5* | Ga14G0362.1 | 823 | 780 | 259 | 1 | 27.204 | 6.076 | -2 |
| *GaAHL23-6* | Ga01G0699.1 | 828 | 828 | 275 | 0 | 28.811 | 6.165 | -2 |
| *GaAHL17-2* | Ga14G1507.1 | 912 | 912 | 303 | 0 | 31.549 | 6.965 | 3 |
| *GaAHL17-6* | Ga05G2337.1 | 906 | 906 | 301 | 0 | 31.475 | 8.539 | 11.5 |
| *GaAHL17-7* | Ga14G0408.1 | 864 | 864 | 287 | 0 | 29.818 | 7.008 | 2.5 |
| *GaAHL17-3* | Ga09G1226.1 | 684 | 684 | 227 | 0 | 24.323 | 7.177 | 2.5 |
| *GaAHL17-4* | Ga09G1261.1 | 681 | 681 | 226 | 0 | 24.08 | 7.11 | 2 |
| *GaAHL17-8* | Ga13G2671.1 | 996 | 996 | 331 | 0 | 33.539 | 10.272 | 8 |
| *GaAHL17-1* | Ga07G1326.1 | 894 | 894 | 297 | 0 | 30.26 | 8.934 | 9.5 |
| *GaAHL17-5* | Ga05G0760.1 | 882 | 882 | 293 | 0 | 30.266 | 8.044 | 8 |
| *GaAHL17-9* | Ga09G0949.1 | 723 | 723 | 240 | 0 | 26.117 | 8.216 | 6 |
| *GaAHL1-1* | Ga01G2662.1 | 2,153 | 990 | 329 | 4 | 33.998 | 10.189 | 9.5 |
| *GaAHL1-2* | Ga08G2062.1 | 3,443 | 1,017 | 338 | 4 | 35.06 | 9.994 | 8.5 |
| *GaAHL1-3* | Ga11G3922.1 | 2,663 | 984 | 327 | 4 | 33.713 | 10.038 | 9.5 |
| *GaAHL7-1* | Ga08G1604.1 | 1,887 | 1,011 | 336 | 4 | 35.001 | 9.423 | 7 |
| *GaAHL7-2* | Ga11G3214.1 | 2,811 | 996 | 331 | 4 | 34.169 | 9.686 | 5.5 |
| *GaAHL3* | Ga12G0146.1 | 2,991 | 1,008 | 335 | 4 | 35.194 | 7.724 | 4 |
| *GaAHL10* | Ga01G1092.1 | 5,343 | 1,095 | 364 | 4 | 37.042 | 10.27 | 13.5 |
| *GaAHL14-1* | Ga11G1047.1 | 5,132 | 1,035 | 344 | 5 | 36.002 | 9.59 | 10.5 |
| *GaAHL14-2* | Ga11G0407.1 | 3,668 | 1,005 | 334 | 6 | 34.953 | 9.668 | 11 |
| *GaAHL14-3* | Ga13G2129.1 | 3,086 | 1,038 | 345 | 5 | 36.364 | 9.054 | 12 |
| *GaAHL13-1* | Ga08G1855.1 | 3,479 | 1,191 | 396 | 4 | 41.39 | 9.894 | 15 |
| *GaAHL13-2* | Ga12G0709.1 | 2,986 | 1,176 | 391 | 4 | 40.562 | 9.945 | 10 |
| *GaAHL5-1* | Ga08G2140.1 | 2,674 | 1,026 | 341 | 4 | 35.443 | 10.638 | 18 |
| *GaAHL5-2* | Ga12G0508.1 | 2,586 | 1,023 | 340 | 4 | 35.293 | 10.376 | 18 |
| *GaAHL9-1* | Ga08G1564.1 | 2,334 | 1,023 | 340 | 4 | 35.041 | 9.669 | 10 |
| *GaAHL9-2* | Ga11G3138.1 | 2,866 | 1,023 | 340 | 4 | 34.974 | 10.556 | 15 |
| *GaAHL9-3* | Ga12G1830.1 | 1,861 | 990 | 329 | 4 | 34.005 | 10.343 | 13 |
| *GaAHL-X1* | Ga01G1578.1 | 4,558 | 1,101 | 366 | 4 | 38.019 | 9.726 | 9.5 |
| *GaAHL-X2* | Ga09G1559.1 | 2,143 | 1,053 | 350 | 4 | 36.202 | 8.62 | 7 |
| *GaAHL-X3* | Ga04G0938.1 | 2,817 | 1,032 | 343 | 4 | 35.308 | 8.791 | 6 |
| *GaAHL-X4* | Ga07G1158.1 | 1,788 | 1,104 | 367 | 4 | 38.831 | 7.114 | 2.5 |
| *GaAHL-X5* | Ga04G1890.1 | 5,580 | 633 | 210 | 4 | 22.774 | 5.37 | -2.5 |

MW, Molecular weight of the amino acid sequence; pI, Isoelectric point.
