# Supplementary material for: Genome-wide identification and analyses of the AHL gene family in cotton (Gossypium)
Source: BMC Genomics. 2020 Jan 22;21:69. doi: 10.1186/s12864-019-6406-6 (PMC6977275; doi:10.1186/s12864-019-6406-6)
Supplement: Supplementary file 2 — Additional file 2. - Information of AHLs in G. hirsutum. a Molecular weight of the amino acid sequence, b Isoelectric point [file 12864_2019_6406_MOESM2_ESM.docx]

**Additional file 2-Information of *AHLs* in *G. hirsutum***

| *Gene Name* | Sequence ID | Gene (bp) | CDS (bp) | Protein (aa) | Intron | MW  (kDa) | pI | Charge |
| --- | --- | --- | --- | --- | --- | --- | --- | --- |
| *Gh-DtAHL22-1* | Gh_D07G1461.1 | 870 | 870 | 289 | 0 | 30.956 | 7.274 | 3 |
| *Gh-DtAHL22-3* | Gh_D11G0864.1 | 900 | 900 | 299 | 0 | 31.401 | 6.512 | 0 |
| *Gh-DtAHL22-2* | Gh_D08G1480.1 | 927 | 927 | 308 | 0 | 32.366 | 6.97 | 2 |
| *Gh-DtAHL24-2* | Gh_D09G1846.1 | 900 | 900 | 299 | 0 | 31.819 | 6.704 | 1.5 |
| *Gh-DtAHL24-1* | Gh_D03G1528.1 | 936 | 936 | 311 | 0 | 33.398 | 6.838 | 3 |
| *Gh-DtAHL24-3* | Gh_D12G2209.1 | 927 | 927 | 308 | 0 | 32.907 | 6.584 | 0.5 |
| *Gh-DtAHL25-1* | Gh_D02G1961.1 | 846 | 846 | 281 | 0 | 28.284 | 9.418 | 5.5 |
| *Gh-DtAHL25-2* | Gh_D04G2013.1 | 846 | 846 | 281 | 0 | 28.405 | 8.982 | 5 |
| *Gh-DtAHL16-1* | Gh_D09G0063.1 | 771 | 771 | 256 | 0 | 27.271 | 9.111 | 7.5 |
| *Gh-DtAHL16-2* | Gh_D11G0652.1 | 759 | 759 | 252 | 0 | 26.805 | 8.113 | 5 |
| *Gh-DtAHL15* | Gh_D10G2301.1 | 933 | 933 | 310 | 0 | 32.506 | 5.728 | -4.5 |
| *Gh-DtAHL20-3* | Gh_D11G2582.1 | 909 | 909 | 302 | 0 | 30.368 | 5.954 | -2 |
| *Gh-DtAHL20-1* | Gh_D02G0418.1 | 888 | 888 | 295 | 0 | 30.459 | 5.349 | -3.5 |
| *Gh-DtAHL20-2* | Gh_D09G2170.1 | 7,788 | 1,173 | 390 | 4 | 41.063 | 6.24 | -2 |
| *Gh-DtAHL23-1* | Gh_D03G1633.1 | 804 | 804 | 267 | 0 | 27.72 | 6.856 | 2 |
| *Gh-DtAHL23-4* | Gh_D12G2080.1 | 801 | 801 | 266 | 0 | 27.943 | 6.739 | 1.5 |
| *Gh-DtAHL23-2* | Gh_D08G1712.1 | 952 | 876 | 291 | 1 | 30.223 | 7.016 | 2.5 |
| *Gh-DtAHL23-3* | Gh_D09G1891.1 | 828 | 828 | 275 | 0 | 28.753 | 6.11 | -2.5 |
| *Gh-DtAHL17-2* | Gh_D02G0859.1 | 909 | 909 | 302 | 0 | 31.515 | 7.168 | 4 |
| *Gh-DtAHL17-6* | Gh_D05G2116.1 | 951 | 951 | 316 | 0 | 33.117 | 8.388 | 12 |
| *Gh-DtAHL17-7* | Gh_Sca005047G03. | 864 | 864 | 287 | 0 | 29.698 | 7.062 | 3 |
| *Gh-DtAHL17-3* | Gh_D09G1016.1 | 684 | 684 | 227 | 0 | 24.102 | 7.234 | 3 |
| *Gh-DtAHL17-4* | Gh_D09G1047.1 | 684 | 684 | 227 | 0 | 24.244 | 6.944 | 2 |
| *Gh-DtAHL17-8* | Gh_D13G2285.1 | 987 | 987 | 328 | 0 | 33.21 | 10.682 | 10 |
| *Gh-DtAHL17-1* | Gh_D07G1189.1 | 894 | 894 | 297 | 0 | 30.348 | 9.49 | 10.5 |
| *Gh-DtAHL17-5* | Gh_D05G0719.1 | 882 | 882 | 293 | 0 | 30.217 | 8.052 | 8.5 |
| *Gh-DtAHL1-1* | Gh_D03G1523.1 | 2,142 | 984 | 327 | 4 | 33.748 | 9.912 | 8.5 |
| *Gh-DtAHL1-2* | Gh_D08G1883.1 | 3,481 | 1,026 | 341 | 4 | 35.321 | 9.669 | 7.5 |
| *Gh-DtAHL1-3* | Gh_D11G0195.1 | 2,653 | 984 | 327 | 4 | 33.742 | 10.038 | 9.5 |
| *Gh-DtAHL7-1* | Gh_D08G1488.1 | 1,962 | 1,011 | 336 | 4 | 34.968 | 9.402 | 6 |
| *Gh-DtAHL7-2* | Gh_D11G0859.1 | 7,568 | 1,128 | 375 | 6 | 39.319 | 10.25 | 12.5 |
| *Gh-DtAHL3* | Gh_D12G2517.1 | 2,996 | 1,008 | 335 | 4 | 35.267 | 8.103 | 5.5 |
| *Gh-DtAHL10* | Gh_D01G0843.1 | 5,339 | 1,095 | 364 | 4 | 37.046 | 10.27 | 14 |
| *Gh-DtAHL14-1* | Gh_D11G2579.1 | 5,134 | 1,035 | 344 | 5 | 35.99 | 9.59 | 10.5 |
| *Gh-DtAHL14-2* | Gh_D11G3027.1 | 3,646 | 1,035 | 344 | 5 | 35.985 | 9.987 | 13 |
| *Gh-DtAHL14-3* | Gh_D13G1808.1 | 3,161 | 1,032 | 343 | 5 | 36.063 | 9.356 | 11.5 |
| *Gh-DtAHL13-1* | Gh_D08G1713.1 | 3,531 | 1,191 | 396 | 4 | 41.386 | 10.202 | 17 |
| *Gh-DtAHL13-2* | Gh_D12G2082.1 | 2,877 | 1,179 | 392 | 4 | 40.516 | 9.063 | 7 |
| *Gh-DtAHL5-1* | Gh_D08G1957.1 | 2,807 | 1,026 | 341 | 4 | 35.479 | 10.638 | 18 |
| *Gh-DtAHL5-2* | Gh_D12G2263.1 | 2,587 | 1,023 | 340 | 4 | 35.256 | 10.307 | 16 |
| *Gh-DtAHL9-1* | Gh_D08G1453.1 | 2,333 | 1,023 | 340 | 4 | 35.066 | 9.853 | 11 |
| *Gh-DtAHL9-2* | Gh_D11G0927.1 | 2,847 | 1,026 | 341 | 4 | 35.075 | 10.487 | 14 |
| *Gh-DtAHL9-3* | Gh_D12G1101.1 | 1,823 | 990 | 329 | 4 | 34.084 | 10.559 | 15 |
| *Gh-DtAHL-X1* | Gh_D01G1264.1 | 4,789 | 1,101 | 366 | 4 | 38.074 | 10.028 | 11 |
| *Gh-DtAHL-X2* | Gh_D09G2438.1 | 2,031 | 1,083 | 360 | 4 | 37.728 | 9.165 | 10.5 |
| *Gh-DtAHL-X3* | Gh_D04G1014.1 | 2,812 | 993 | 330 | 4 | 33.612 | 8.456 | 4 |
| *Gh-DtAHL-X4* | Gh_D07G1049.1 | 1,689 | 1,095 | 364 | 4 | 38.227 | 7.851 | 4.5 |
| *Gh-DtAHL-X5* | Gh_D04G0182.1 | 5,573 | 633 | 210 | 4 | 22.753 | 6.258 | -0.5 |
| *Gh-AtAHL22-1* | Gh_A07G1349.1 | 870 | 870 | 289 | 0 | 30.964 | 8.252 | 4.5 |
| *Gh-AtAHL22-3* | Gh_A11G0743.1 | 906 | 906 | 301 | 0 | 31.636 | 6.512 | 0 |
| *Gh-AtAHL22-2* | Gh_A08G1196.1 | 921 | 921 | 306 | 0 | 32.168 | 6.97 | 2 |
| *Gh-AtAHL24-2* | Gh_A09G1737.1 | 900 | 900 | 299 | 0 | 31.884 | 6.753 | 2 |
| *Gh-AtAHL24-1* | Gh_A03G0105.1 | 930 | 930 | 309 | 0 | 33.034 | 6.93 | 3.5 |
| *Gh-AtAHL24-3* | Gh_A12G2031.1 | 924 | 924 | 307 | 0 | 32.778 | 6.584 | 0.5 |
| *Gh-AtAHL25-1* | Gh_A03G1494.1 | 849 | 849 | 282 | 0 | 28.458 | 8.693 | 4.5 |
| *Gh-AtAHL25-2* | Gh_A04G1423.1 | 849 | 849 | 282 | 0 | 28.476 | 8.68 | 5 |
| *Gh-AtAHL16-1* | Gh_A09G0066.1 | 771 | 771 | 256 | 0 | 27.273 | 8.707 | 6.5 |
| *Gh-AtAHL16-2* | Gh_Sca009301G01. | 759 | 759 | 252 | 0 | 26.822 | 8.486 | 6 |
| *Gh-AtAHL15* | Gh_A10G1993.1 | 933 | 933 | 310 | 0 | 32.541 | 5.897 | -4 |
| *Gh-AtAHL20-3* | Gh_A11G2276.1 | 909 | 909 | 302 | 0 | 30.377 | 5.954 | -2 |
| *Gh-AtAHL20-1* | Gh_A02G0359.1 | 888 | 888 | 295 | 0 | 30.471 | 5.349 | -3.5 |
| *Gh-AtAHL20-2* | Gh_A09G1967.1 | 6970 | 1272 | 423 | 6 | 45.291 | 8.769 | 9 |
| *Gh-AtAHL23-1* | Gh_A03G0019.1 | 804 | 804 | 267 | 0 | 27.865 | 7.07 | 3 |
| *Gh-AtAHL23-4* | Gh_A12G1899.1 | 801 | 801 | 266 | 0 | 27.957 | 6.912 | 2.5 |
| *Gh-AtAHL23-2* | Gh_A08G1420.1 | 864 | 864 | 287 | 0 | 29.755 | 6.6 | 0.5 |
| *Gh-AtAHL23-3* | Gh_A09G2350.1 | 804 | 744 | 247 | 1 | 25.789 | 8.056 | 2 |
| *Gh-AtAHL23-5* | Gh_A09G2336.1 | 828 | 828 | 275 | 0 | 28.975 | 6.507 | 0 |
| *Gh-AtAHL17-2* | Gh_A02G0807.1 | 909 | 909 | 302 | 0 | 31.465 | 7.439 | 5 |
| *Gh-AtAHL17-6* | Gh_A05G1881.1 | 957 | 957 | 318 | 0 | 33.433 | 8.533 | 12.5 |
| *Gh-AtAHL17-6d* | Gh_A05G1882.1 | 570 | 570 | 189 | 0 | 19.244 | 7.559 | 2.5 |
| *Gh-AtAHL17-7* | Gh_A06G1884.1 | 864 | 864 | 287 | 0 | 29.826 | 7.062 | 3 |
| *Gh-AtAHL17-3* | Gh_A09G0996.1 | 684 | 684 | 227 | 0 | 24.324 | 6.871 | 1.5 |
| *Gh-AtAHL17-4* | Gh_A09G1026.1 | 681 | 681 | 226 | 0 | 24.125 | 6.775 | 1 |
| *Gh-AtAHL17-8* | Gh_A13G1898.1 | 996 | 996 | 331 | 0 | 33.544 | 10.478 | 8.5 |
| *Gh-AtAHL17-1* | Gh_A07G1089.1 | 894 | 894 | 297 | 0 | 30.272 | 8.934 | 9.5 |
| *Gh-AtAHL17-5* | Gh_A05G0589.1 | 882 | 882 | 293 | 0 | 30.323 | 7.82 | 7 |
| *Gh-AtAHL17-9* | Gh_A09G0757.1 | 480 | 480 | 159 | 0 | 17.227 | 8.085 | 3.5 |
| *Gh-AtAHL1-1* | Gh_A03G0117.1 | 2176 | 990 | 329 | 4 | 33.898 | 10.059 | 8.5 |
| *Gh-AtAHL1-2* | Gh_A08G1576.1 | 4570 | 1017 | 338 | 4 | 35.09 | 9.994 | 8.5 |
| *Gh-AtAHL1-3* | Gh_A11G0184.1 | 2667 | 984 | 327 | 4 | 33.768 | 10.166 | 10.5 |
| *Gh-AtAHL7-1* | Gh_A08G1204.1 | 1869 | 993 | 330 | 4 | 34.543 | 8.695 | 5 |
| *Gh-AtAHL7-2* | Gh_A11G0739.1 | 2707 | 996 | 331 | 4 | 34.33 | 10.05 | 7.5 |
| *Gh-AtAHL3* | Gh_A12G2390.1 | 3005 | 1008 | 335 | 4 | 35.194 | 7.724 | 4 |
| *Gh-AtAHL10* | Gh_A01G0816.1 | 5301 | 1095 | 364 | 4 | 37.116 | 10.368 | 14.5 |
| *Gh-AtAHL14-1* | Gh_A11G2272.1 | 5289 | 1035 | 344 | 5 | 36.006 | 9.59 | 10.5 |
| *Gh-AtAHL14-2* | Gh_A11G2664.1 | 10972 | 1035 | 344 | 5 | 35.918 | 10.042 | 13 |
| *Gh-AtAHL14-3* | Gh_A13G1478.1 | 3089 | 1038 | 345 | 5 | 36.388 | 7.939 | 9.5 |
| *Gh-AtAHL13-1* | Gh_A08G1421.1 | 3476 | 1191 | 396 | 4 | 41.348 | 9.894 | 15 |
| *Gh-AtAHL13-2* | Gh_A12G1901.1 | 2986 | 1176 | 391 | 4 | 40.577 | 9.945 | 10 |
| *Gh-AtAHL5-1* | Gh_A08G2384.1 | 2674 | 1026 | 341 | 4 | 35.429 | 10.638 | 18 |
| *Gh-AtAHL5-2* | Gh_A12G2087.1 | 2584 | 1023 | 340 | 4 | 35.231 | 10.544 | 19 |
| *Gh-AtAHL9-1* | Gh_A08G1169.1 | 2334 | 1023 | 340 | 4 | 35.011 | 9.669 | 10 |
| *Gh-AtAHL9-2* | Gh_A11G3057.1 | 2704 | 1026 | 341 | 4 | 35.102 | 10.556 | 15 |
| *Gh-AtAHL9-3* | Gh_A12G0982.1 | 1860 | 990 | 329 | 4 | 34.062 | 10.343 | 13 |
| *Gh-AtAHL-X1* | Gh_A01G1159.1 | 3869 | 990 | 329 | 3 | 34.293 | 9.767 | 7.5 |
| *Gh-AtAHL-X2* | Gh_A09G2445.1 | 2143 | 1053 | 350 | 4 | 36.17 | 8.621 | 7.5 |
| *Gh-AtAHL-X3* | Gh_A04G0557.1 | 2836 | 1050 | 349 | 4 | 35.9 | 8.451 | 5 |
| *Gh-AtAHL-X4* | Gh_A07G0971.1 | 1789 | 1095 | 364 | 4 | 38.26 | 7.47 | 3.5 |
| *Gh-AtAHLX-5* | Gh_A05G3407.1 | 5527 | 633 | 210 | 4 | 22.942 | 5.774 | -1.5 |

MW, Molecular weight of the amino acid sequence; pI, Isoelectric point.
