# Supplementary material for: Genome-wide identification and analyses of the AHL gene family in cotton (Gossypium)
Source: BMC Genomics. 2020 Jan 22;21:69. doi: 10.1186/s12864-019-6406-6 (PMC6977275; doi:10.1186/s12864-019-6406-6)
Supplement: Supplementary file 3 — Additional file 3. - The orthologous relationship and type of AHL proteins in V. vinifera, T. cacao, A.thaliana and G. raimondii. The forms in pink indicated the Type-I AHL genes, those in yellow indicated the Type-III AHL genes and those in blue indicated the Type-II AHL genes. The lines repented the loss of orthologous gene [file 12864_2019_6406_MOESM3_ESM.docx]

**Additional file 3**

**The orthologous relationship and type of AHL proteins in *V. vinifera, T. cacao, A.thaliana* and *G. raimondii***

| **AtAHL** | **VvAHL** | | **TcAHL** | **GrAHL** |  |  |
| --- | --- | --- | --- | --- | --- | --- |
| *AHL22* | GSVIVT01027625001 | | Thecc1EG005492 | Gorai.001G173500 |  |  |
|  |  |  |  | Gorai.007G091800 |  |  |
|  |  |  |  | Gorai.004G160700 |  |  |
| *AHL24* | GSVIVT01019932001 | | Thecc1EG016102 | Gorai.006G211500 |  |  |
|  |  |  |  | Gorai.003G167700 |  |  |
|  |  |  |  | Gorai.008G240700 |  |  |
| *AHL16* | —— | | Thecc1EG001878 | Gorai.006G007800 |  |  |
|  |  |  |  | Gorai.007G070000 |  |  |
| *AHL25* | GSVIVT01013438001 | | Thecc1EG007603 | Gorai.005G215400 |  |  |
|  |  |  | Thecc1EG034810 | Gorai.012G138000 |  |  |
| *AHL15* | GSVIVT01016497001 | | Thecc1EG042731 | Gorai.011G267800 |  |  |
| *AHL20* | GSVIVT01036210001 | | Thecc1EG020823 | Gorai.007G280400 |  |  |
|  |  |  | Thecc1EG026251 | Gorai.005G048000 |  |  |
|  |  |  |  | Gorai.006G247900 |  |  |
| *AHL23* | GSVIVT01013202001 | | Thecc1EG015730 | Gorai.003G181200 |  |  |
|  |  |  |  | Gorai.008G226900 |  |  |
|  |  |  |  | Gorai.004G185900 |  |  |
|  |  |  |  | Gorai.006G216300 |  |  |
| *AHL17* | ——— | | Thecc1EG034495 | Gorai.005G096700 |  |  |
|  |  |  |  | Gorai.009G230300 |  |  |
|  |  |  |  | Gorai.010G035300 |  |  |
|  |  |  |  | Gorai.006G120100 |  |  |
|  |  |  | Thecc1EG040931 | Gorai.006G124100 |  |  |
|  |  |  |  | Gorai.013G253800 |  |  |
|  |  |  |  | Gorai.001G133900 |  |  |
|  |  |  |  | Gorai.009G075100 |  |  |
|  | *AHL14* | | | GSVIVT01016497001 | Thecc1EG042731 | Gorai.011G267800 |
|  |  |  |  |  |  | Gorai.013G186600 |
|  |  |  |  |  |  | Gorai.007G280000 |
|  | *AHL1* | | | GSVIVT01019937001 | Thecc1EG016097 | Gorai.003G167100 |
|  |  |  |  |  |  | Gorai.007G021700 |
|  |  |  |  |  |  | Gorai.004G203700 |
|  | *AHL7* | | | GSVIVT01027617001 | Thecc1EG005503 | Gorai.007G091400 |
|  |  |  |  |  |  | Gorai.004G161300 |
|  | *AHL3* | | | GSVIVT01018513001 | Thecc1EG014987 | Gorai.008G283600 |
|  | *AHL10* | | | GSVIVT01013426001 | Thecc1EG034823 | Gorai.002G112700 |
|  | *AHL13* | | | GSVIVT01013200001 | Thecc1EG015732 | Gorai.008G227100 |
|  |  |  |  |  |  | Gorai.004G186000 |
|  | *AHL9* | | | GSVIVT01026888001 | Thecc1EG005351 | Gorai.004G158000 |
|  |  |  |  |  |  | Gorai.008G122100 |
|  |  |  |  |  |  | Gorai.007G098600 |
|  | *AHL5* | | | GSVIVT01019763001 | Thecc1EG016243 | Gorai.008G246700 |
|  |  |  |  |  |  | Gorai.004G211500 |
|  | ——— | | | GSVIVT01033765001 | Thecc1EG022420 | Gorai.006G158700 |
|  |  |  |  |  |  | Gorai.001G119100 |
|  | ——— | | | ——— | Thecc1EG041524 | Gorai.002G160000 |
|  |  |  |  |  |  | Gorai.009G408800 |
|  |  |  |  |  |  | Gorai.012G024700 |

Note:The forms in pink indicated the Type-I AHL genes, those in yellow indicated the Type-III AHL genes and those in blue indicated the Type-II AHL genes. The lines repented the loss of orthologous gene.
