# Supplementary figures and images for: Genome-wide identification and analyses of the AHL gene family in cotton (Gossypium)
Source: BMC Genomics. 2020 Jan 22;21:69. doi: 10.1186/s12864-019-6406-6 (PMC6977275; doi:10.1186/s12864-019-6406-6)

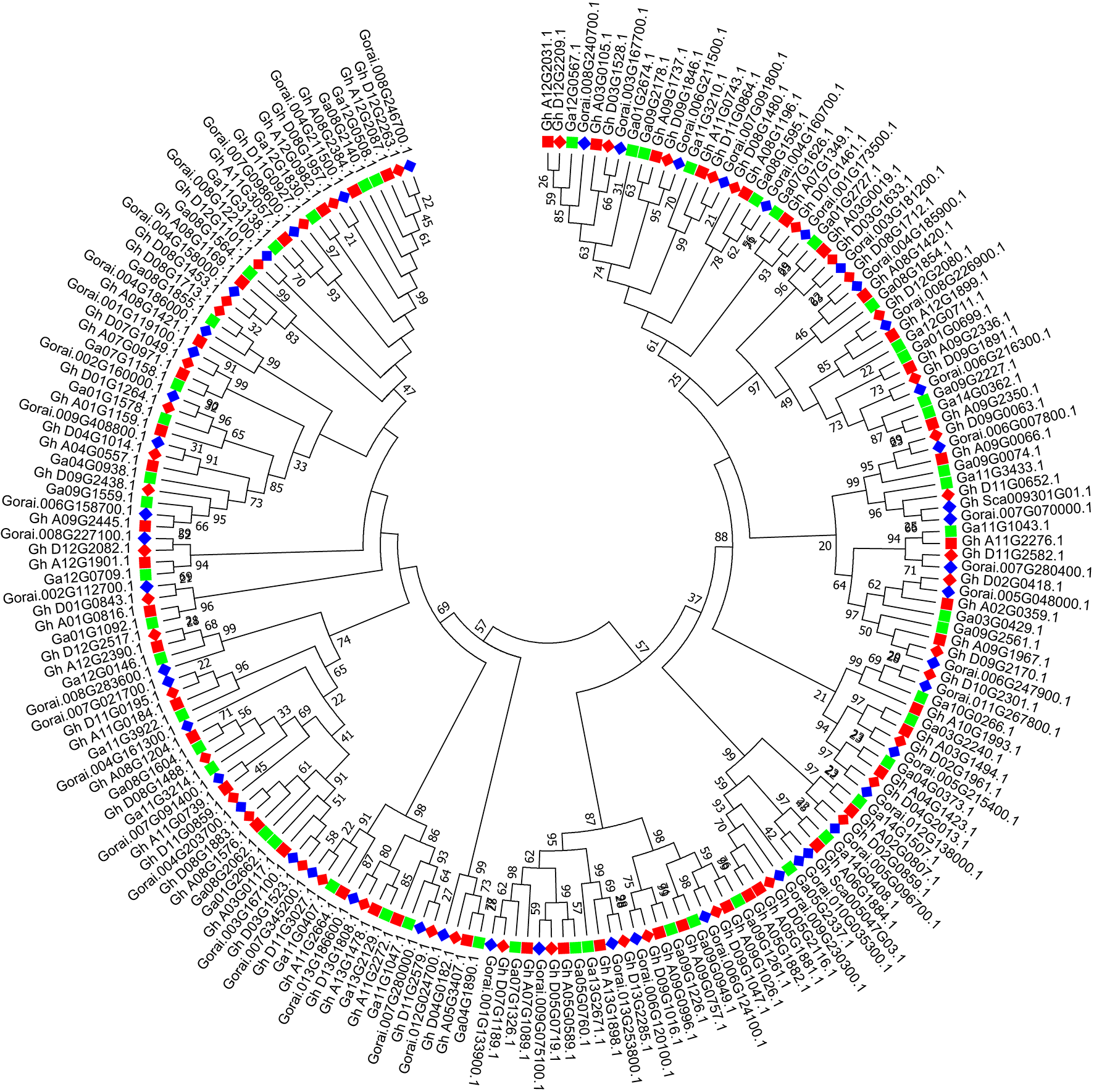

Supplement: Supplementary file 4 — Additional file 4. - Phylogenetic relationship of AHL proteins in cotton. AHL proteins from G. raimondii, G. arboreum and G. hirsutum are marked with blue rhombus, green squares, and red rhombus squares, respectively [file 12864_2019_6406_MOESM4_ESM.tif]

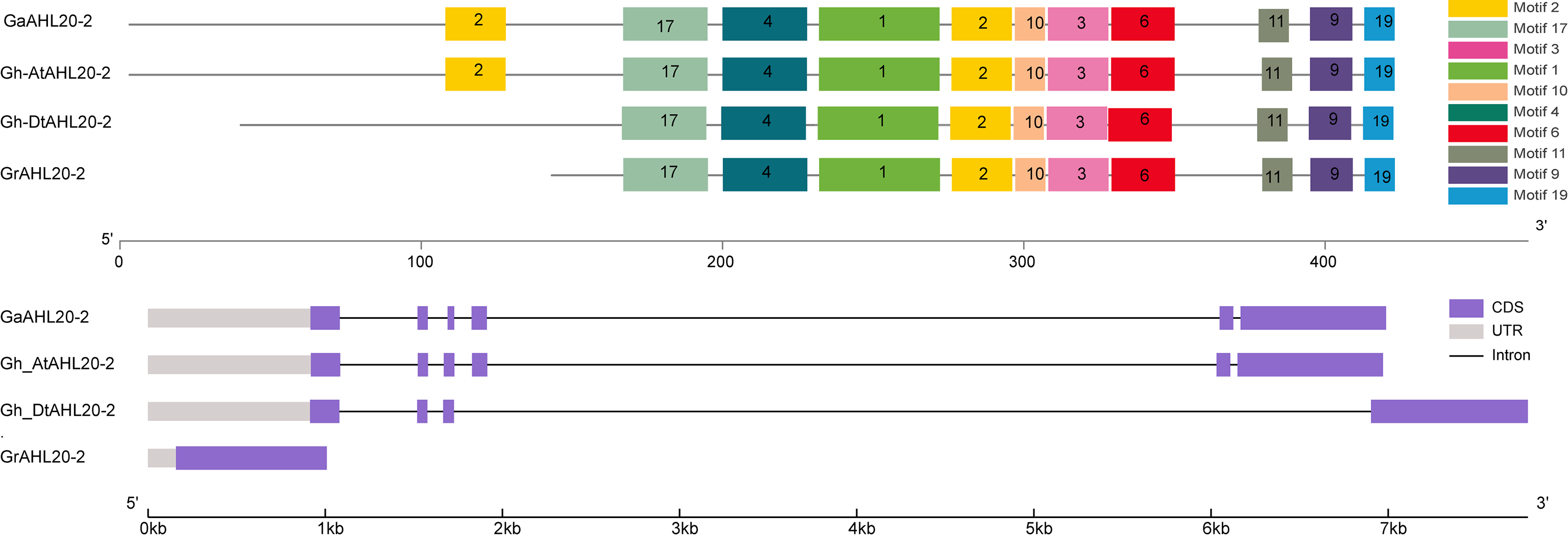

Supplement: Supplementary file 5 — Additional file 5. - The variations of gene structures and motifs of AHL20–2 from G. raimondii, G. arboreum and G. hirsutum. Gene structure and conserved motifs were predicted from the GSDS and MEME website. The length of proteins and DNA sequence was estimated using the scale at the bottom. The motifs were displayed in different colored boxes with Arabic numerals; black line indicated the non-conserved amino acid or intron. Gray boxes indicate untranslated 5- and 3-regions, blue boxes indicate exons. The sequences of motifs were listed in additional file 6 [file 12864_2019_6406_MOESM5_ESM.tif]

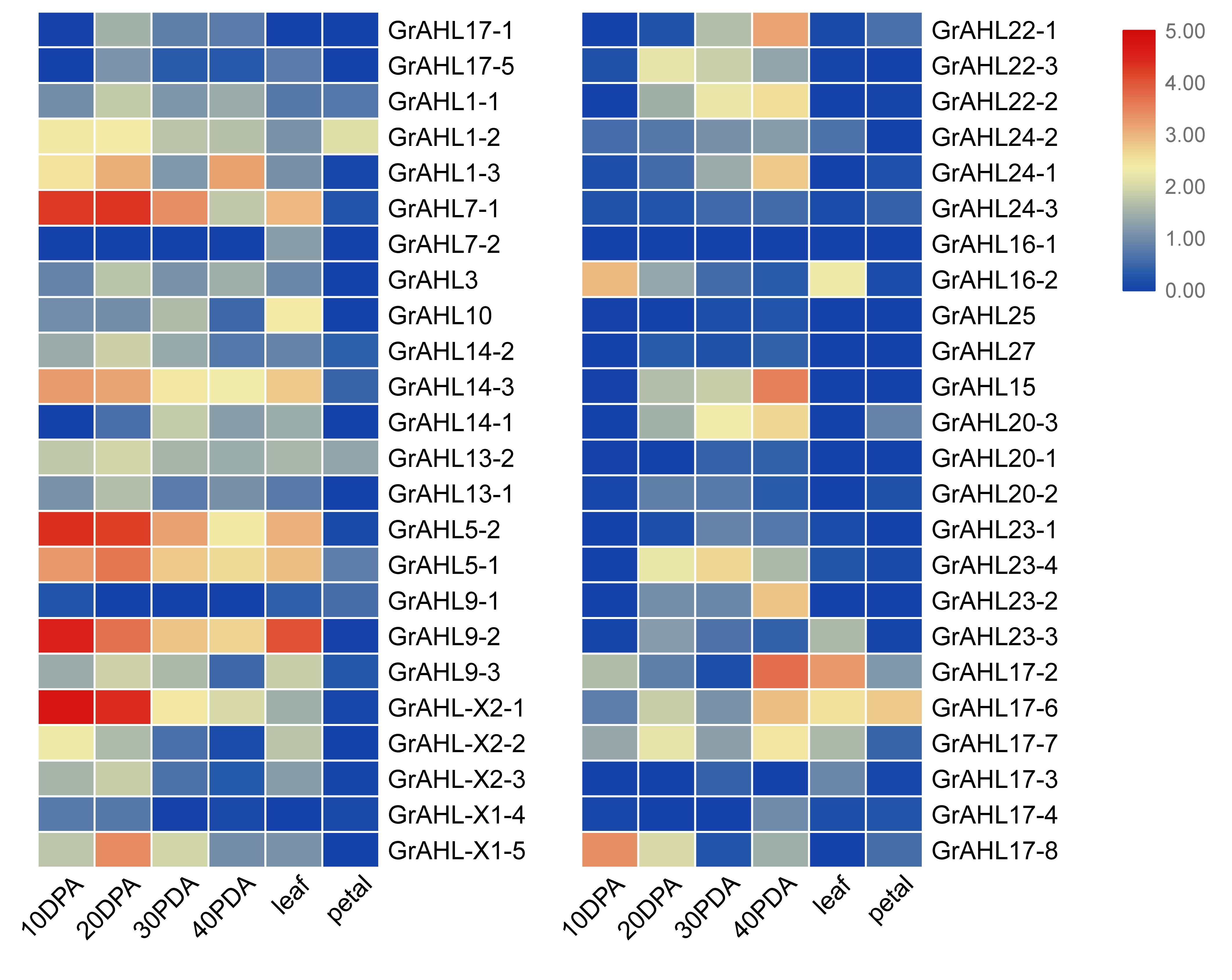

Supplement: Supplementary file 6 — Additional file 6. - The sequences of motifs predicted by MEME (http://meme-suite.org/tools/meme) [file 12864_2019_6406_MOESM6_ESM.tif]

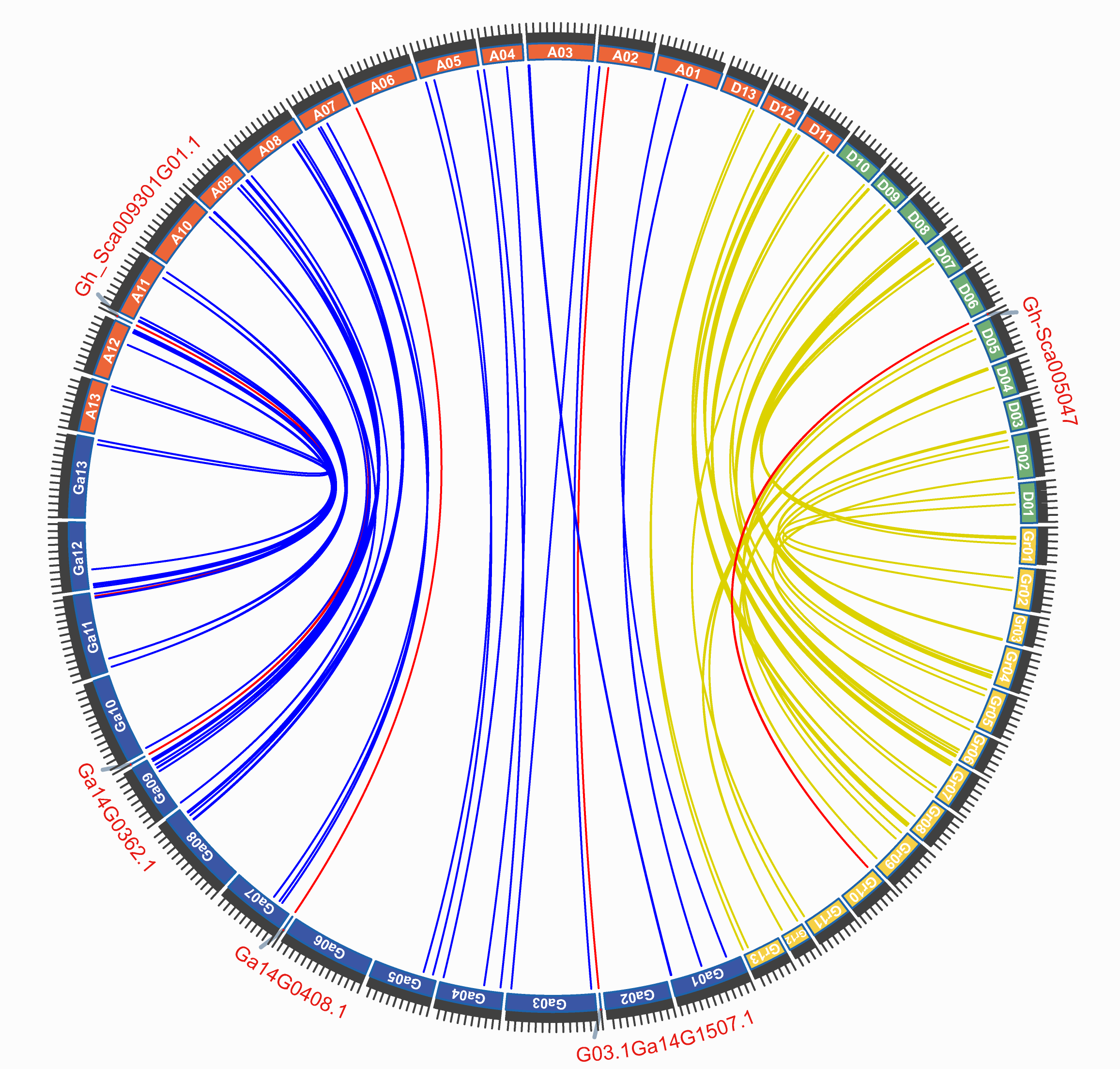

Supplement: Supplementary file 7 — Additional file 7. - The expression profiles of GrAHLs. The heatmap was generated on the basis of RNA-seq data from the website (http://structuralbiology.cau.edu.cn/gossypium), the color scale was shown at the right. Higher expression levels were shown in red, and lower in blue. DPA represented the day of ovule after anthesis [file 12864_2019_6406_MOESM7_ESM.tif]

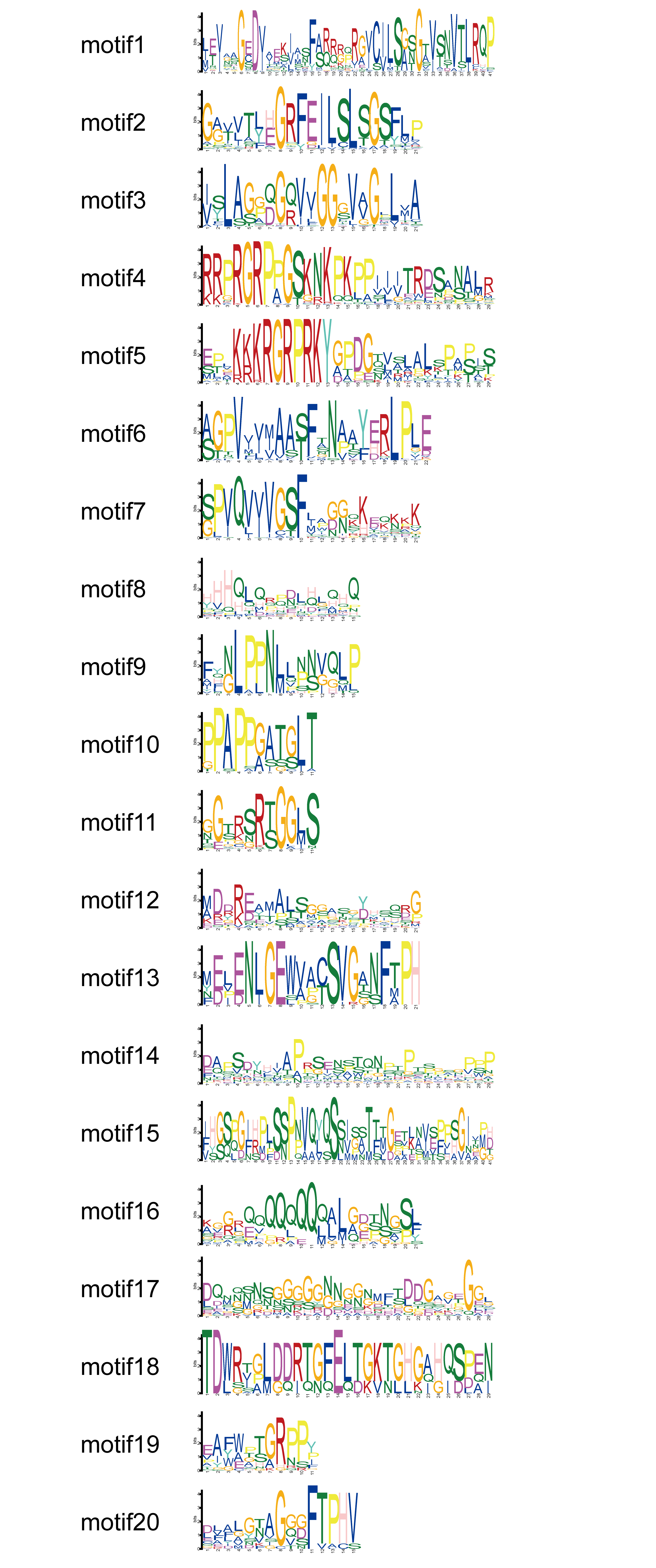

Supplement: Supplementary file 8 — Additional file 8. - The circos map of AHLs in G. raimondii, G. arbretum and G. hirsutum.The collinearity of AHL genes between G. raimondii and D-subgenome in G. hirsutum were showed in orange lines, that between G. arbretum and the A-subgenome in G. hirsutum in blue lines. AHL genes located in scaffolds were showed in red lines, and the locations of scaffolds were putatived [file 12864_2019_6406_MOESM8_ESM.tif]
