# Supplementary material for: Genome-wide identification and analyses of the AHL gene family in cotton (Gossypium)
Source: BMC Genomics. 2020 Jan 22;21:69. doi: 10.1186/s12864-019-6406-6 (PMC6977275; doi:10.1186/s12864-019-6406-6)
Supplement: Supplementary file 9 — Additional file 9. - Ka, Ks and Ka/Ks ratio between orthologous genes pairs from G. raimondii and D-subgenome in G. hirsutum [file 12864_2019_6406_MOESM9_ESM.docx]

**Additional file 9**

**Ka, Ks and Ka/Ks ratio between orthologous genes pairs from *G.raimondii* and D-subgenome in *G.hirsutum***

| AHL  Name | GrAHLid | Gh_DtAHLid | Ka | Ks | Ka/Ks | P-Value |
| --- | --- | --- | --- | --- | --- | --- |
| *AHL22-1* | Gorai.001G173500.1 | Gh_D07G1461.1 | 1.03822 | 0.882287 | 1.17674 | 0.0020379 |
| *AHL22-3* | Gorai.007G091800.1 | Gh_D11G0864.1 | 1.04027 | 0.88498 | 1.17547 | 0.0004859 |
| *AHL22-2* | Gorai.004G160700.1 | Gh_D08G1480.1 | 1.05632 | 0.840312 | 1.25705 | 7.42E-06 |
| *AHL24-2* | Gorai.006G211500.1 | Gh_D09G1846.1 | 1.03369 | 0.903226 | 1.14444 | 0.0048893 |
| *AHL24-1* | Gorai.003G167700.1 | Gh_D03G1528.1 | 0.98754 | 1.04632 | 0.943827 | 4.48E-01 |
| *AHL24-3* | Gorai.008G240700.1 | Gh_D12G2209.1 | 1.00499 | 0.985544 | 1.01973 | 0.638419 |
| *AHL16-1* | Gorai.006G007800.1 | Gh_D09G0063.1 | 1.04248 | 0.854582 | 1.21987 | 0.0002720 |
| *AHL16-2* | Gorai.007G070000.1 | Gh_D11G0652.1 | 1.05663 | 0.797976 | 1.32414 | 3.93E-06 |
| *AHL25-1* | Gorai.005G215400.1 | Gh_D02G1961.1 | 1.03678 | 0.905647 | 1.14479 | 0.0039164 |
| *AHL25-2* | Gorai.012G138000.1 | Gh_D04G2013.1 | 1.06033 | 0.842729 | 1.25821 | 1.53E-06 |
| *AHL15* | Gorai.011G267800.1 | Gh_D10G2301.1 | 1.01827 | 0.944515 | 1.07809 | 0.105103 |
| *AHL20-3* | Gorai.007G280400.1 | Gh_D11G2582.1 | 1.01692 | 0.950289 | 1.07011 | 0.140859 |
| *AHL20-1* | Gorai.005G048000.1 | Gh_D02G0418.1 | 1.02152 | 0.934187 | 1.09349 | 0.0438809 |
| *AHL20-2* | Gorai.006G247900.1 | Gh_D09G2170.1 | 1.03907 | 0.886915 | 1.17156 | 0.0023549 |
| *AHL23-1* | Gorai.003G181200.1 | Gh_D03G1633.1 | 0.99187 | 1.02701 | 0.965796 | 0.465814 |
| *AHL23-4* | Gorai.008G226900.1 | Gh_D12G2080.1 | 1.01978 | 0.941918 | 1.08267 | 0.151396 |
| *AHL23-2* | Gorai.004G185900.1 | Gh_D08G1712.1 | 1.08213 | 0.795064 | 1.36106 | 2.16E-05 |
| *AHL23-3* | Gorai.006G216300.1 | Gh_D09G1891.1 | 1.00222 | 0.993926 | 1.00834 | 0.849404 |
| *AHL17-2* | Gorai.005G096700.1 | Gh_D02G0859.1 | 0.98728 | 1.03603 | 0.95295 | 0.280025 |
| *AHL17-6* | Gorai.009G230300.1 | Gh_D05G2116.1 | 1.0086 | 0.973746 | 1.03579 | 0.415828 |
| *AHL17-7* | Gorai.010G035300.1 | Gh_Sca005047G03.1 | 1.02036 | 0.940352 | 1.08509 | 0.0853898 |
| *AHL17-3* | Gorai.006G120100.1 | Gh_D09G1016.1 | 0.97274 | 1.08921 | 0.893074 | 0.0382102 |
| *AHL17-4* | Gorai.006G124100.1 | Gh_D09G1047.1 | 0.97493 | 1.08949 | 0.894852 | 0.0329006 |
| *AHL17-8* | Gorai.013G253800.1 | Gh_D13G2285.1 | 1.01072 | 0.967023 | 1.04519 | 0.346983 |
| *AHL17-1* | Gorai.001G133900.1 | Gh_D07G1189.1 | 1.01143 | 0.963817 | 1.0494 | 0.356353 |
| *AHL17-5* | Gorai.009G075100.1 | Gh_D05G0719.1 | 0.98980 | 1.0322 | 0.958927 | 0.396284 |
| *AHL1-1* | Gorai.003G167100.1 | Gh_D03G1523.1 | 1.02122 | 0.933942 | 1.09346 | 0.0377111 |
| *AHL1-2* | Gorai.004G203700.1 | Gh_D08G1883.1 | 1.02914 | 0.904575 | 1.1377 | 0.0035086 |
| *AHL1-3* | Gorai.007G021700.1 | Gh_D11G0195.1 | 1.00024 | 0.999244 | 1.00099 | 1.0 |
| *AHL7-1* | Gorai.004G161300.1 | Gh_D08G1488.1 | 0.97897 | 1.07304 | 0.912339 | 0.0394591 |
| *AHL7-2* | Gorai.007G091400.1 | Gh_D11G0859.1 | 1.01142 | 0.960143 | 1.05341 | 0.23719 |
| *AHL3* | Gorai.008G283600.1 | Gh_D12G2517.1 | 0.98847 | 1.03948 | 0.950927 | 0.248284 |
| *AHL10* | Gorai.002G112700.1 | Gh_D01G0843.1 | 1.03069 | 0.905702 | 1.138 | 0.0031456 |
| *AHL14-1* | Gorai.007G280000.1 | Gh_D11G2579.1 | 1.01496 | 0.947881 | 1.07077 | 0.123702 |
| *AHL14-2* | Gorai.007G345200.1 | Gh_D11G3027.1 | 1.0278 | 0.90896 | 1.13074 | 0.0053221 |
| *AHL14-3* | Gorai.013G186600.1 | Gh_D13G1808.1 | 1.01695 | 0.939676 | 1.08223 | 0.0764892 |
| *AHL13-1* | Gorai.004G186000.1 | Gh_D08G1713.1 | 1.03567 | 0.885726 | 1.16929 | 0.0004413 |
| *AHL13-2* | Gorai.008G227100.1 | Gh_D12G2082.1 | 1.01829 | 0.942982 | 1.07986 | 0.0557207 |
| *AHL5-1* | Gorai.004G211500.1 | Gh_D08G1957.1 | 0.99859 | 1.00479 | 0.993836 | 0.854046 |
| *AHL5-2* | Gorai.008G246700.1 | Gh_D12G2263.1 | 1.01428 | 0.953956 | 1.06324 | 0.154757 |
| *AHL9-1* | Gorai.004G158000.1 | Gh_D08G1453.1 | 1.03925 | 0.878616 | 1.18283 | 0.000267 |
| *AHL9-2* | Gorai.007G098600.1 | Gh_D11G0927.1 | 1.04048 | 0.872213 | 1.19292 | 0.000205 |
| *AHL9-3* | Gorai.008G122100.1 | Gh_D12G1101.1 | 1.02698 | 0.917235 | 1.11964 | 0.016623 |
| *AHLx-1* | Gorai.002G160000.1 | Gh_D01G1264.1 | 1.00914 | 0.966949 | 1.04363 | 0.343876 |
| *AHLX-2* | Gorai.006G158700.1 | Gh_D09G2438.1 | 0.99556 | 1.01622 | 0.979671 | 0.649961 |
| *AHLx-3* | Gorai.009G408800.1 | Gh_D04G1014.1 | 0.99057 | 1.03293 | 0.958995 | 0.354696 |
| *AHLx-4* | Gorai.001G119100.1 | Gh_D07G1049.1 | 0.98217 | 1.0641 | 0.923011 | 0.0608334 |
| *AHLx-5* | Gorai.012G024700.1 | Gh_D04G0182.1 | 1.03948 | 0.84009 | 1.23734 | 0.0012141 |
