# Supplementary material for: Genome-wide identification and analyses of the AHL gene family in cotton (Gossypium)
Source: BMC Genomics. 2020 Jan 22;21:69. doi: 10.1186/s12864-019-6406-6 (PMC6977275; doi:10.1186/s12864-019-6406-6)
Supplement: Supplementary file 10 — Additional file 10. - Ka, Ks and Ka/Ks ratio between orthologous gene pairs from G. arbretum and A-subgenome of G. hirsutum [file 12864_2019_6406_MOESM10_ESM.docx]

**Additional file 10**

**Ka, Ks and Ka/Ks ratio between orthologous gene pairs from *G.arbretum* and A-subgenome of *G.hirsutum***

| AHL Name | Gh_AtAHLid | GaAHLid | Ka | Ks | Ka/Ks | P-Value |
| --- | --- | --- | --- | --- | --- | --- |
| *AHL22-1* | Gh_A07G1349.1 | Ga07G1626.1 | 0.998106 | 1.00515 | 0.99299 | 0.871873 |
| *AHL22-3* | Gh_A11G0743.1 | Ga11G3210.1 | 1.0001 | 0.999739 | 1.00036 | 1 |
| *AHL22-2* | Gh_A08G1196.1 | Ga08G1595.1 | 0.975361 | 1.06404 | 0.916654 | 0.0542319 |
| *AHL24-2* | Gh_A09G1737.1 | Ga09G2178.1 | 0.992717 | 1.02032 | 0.972946 | 0.573494 |
| *AHL24-1* | Gh_A03G0105.1 | Ga01G2674.1 | 0.923396 | 1.26149 | 0.731987 | 9.63E-09 |
| *AHL24-3* | Gh_A12G2031.1 | Ga12G0567.1 | 0.91699 | 1.23988 | 0.739581 | 1.02E-09 |
| *AHL16-1* | Gh_A09G0066.1 | Ga09G0074.1 | 0.9849 | 1.04386 | 0.943517 | 0.204305 |
| *AHL16-2* | Gh_Sca009301G01.1 | Ga11G3433.1 | 1.02659 | 0.929119 | 1.10491 | 0.0741557 |
| *AHL25-1* | Gh_A03G1494.1 | Ga03G2240.1 | 0.954863 | 1.10686 | 0.862674 | 0.00264433 |
| *AHL25-2* | Gh_A04G1423.1 | Ga04G0373.1 | 1.02733 | 0.936453 | 1.09704 | 0.093955 |
| *AHL15* | Gh_A10G1993.1 | Ga10G0266.1 | 0.961926 | 1.13324 | 0.848831 | 0.00191919 |
| *AHL20-3* | Gh_A11G2276.1 | Ga11G1043.1 | 0.989623 | 1.02795 | 0.962713 | 0.476376 |
| *AHL20-1* | Gh_A02G0359.1 | Ga03G0429.1 | 1.01503 | 0.961178 | 1.05603 | 0.251045 |
| *AHL20-2* | Gh_A09G1967.1 | Ga09G2561.1 | 0.993789 | 1.01847 | 0.97577 | 0.567688 |
| *AHL23-1* | Gh_A03G0019.1 | Ga01G2727.1 | 0.915553 | 1.30387 | 0.70218 | 3.94E-12 |
| *AHL23-4* | Gh_A12G1899.1 | Ga12G0711.1 | 0.92682 | 1.20978 | 0.766107 | 2.47E-07 |
| *AHL23-2* | Gh_A08G1420.1 | Ga08G1854.1 | 1.01741 | 0.948897 | 1.0722 | 0.124595 |
| *AHL23-3* | Gh_A09G2350.1 | Ga09G2227.1 | 0.966312 | 1.09067 | 0.885979 | 0.016026 |
| *AHL23-5* | Gh_A09G2336.1 | Ga14G0362.1 | 1.01081 | 0.968721 | 1.04344 | 0.521243 |
| *AHL17-2* | Gh_A02G0807.1 | Ga14G1507.1 | 1.0256 | 0.928517 | 1.10455 | 0.0657385 |
| *AHL17-6* | Gh_A05G1881.1 | Ga05G2337.1 | 0.96342 | 1.10734 | 0.870034 | 0.0048772 |
| *AHL17-7* | Gh_A06G1884.1 | Ga14G0408.1 | 0.984671 | 1.05735 | 0.931259 | 0.239266 |
| *AHL17-3* | Gh_A09G0996.1 | Ga09G1226.1 | 0.962218 | 1.12514 | 0.855196 | 0.0086738 |
| *AHL17-4* | Gh_A09G1026.1 | Ga09G1261.1 | 0.996398 | 1.01132 | 0.985241 | 0.83997 |
| *AHL17-8* | Gh_A13G1898.1 | Ga13G2671.1 | 1.03866 | 0.899268 | 1.15501 | 0.0048985 |
| *AHL17-1* | Gh_A07G1089.1 | Ga07G1326.1 | 0.97204 | 1.1 | 0.883671 | 0.0038815 |
| *AHL17-5* | Gh_A05G0589.1 | Ga05G0760.1 | 0.971431 | 1.08685 | 0.893805 | 0.0270504 |
| *AHL17-9* | Gh_A09G0757.1 | Ga09G0949.1 | 1.0011 | 0.996205 | 1.00491 | 0.926648 |
| *AHL1-1* | Gh_A03G0117.1 | Ga01G2662.1 | 1.0046 | 0.989106 | 1.01567 | 0.76497 |
| *AHL1-2* | Gh_A08G1576.1 | Ga08G2062.1 | 0.960171 | 1.12043 | 0.856963 | 0.0001427 |
| *AHL1-3* | Gh_A11G0184.1 | Ga11G3922.1 | 1.00137 | 0.996648 | 1.00474 | 0.940497 |
| *AHL7-1* | Gh_A08G1204.1 | Ga08G1604.1 | 0.971416 | 1.07017 | 0.907719 | 0.052679 |
| *AHL7-2* | Gh_A11G0739.1 | Ga11G3214.1 | 0.944057 | 1.1566 | 0.816233 | 1.24E-06 |
| *AHL3* | Gh_A12G2390.1 | Ga12G0146.1 | 0.99294 | 1.02095 | 0.972567 | 0.56947 |
| *AHL10* | Gh_A01G0816.1 | Ga01G1092.1 | 0.978321 | 1.05642 | 0.926073 | 0.074365 |
| *AHL14-1* | Gh_A11G2272.1 | Ga11G1047.1 | 1.01473 | 0.956914 | 1.06042 | 0.201683 |
| *AHL14-2* | Gh_A11G2664.1 | Ga11G0407.1 | 1.01691 | 0.948576 | 1.07204 | 0.169087 |
| *AHL14-3* | Gh_A13G1478.1 | Ga13G2129.1 | 1.01082 | 0.964912 | 1.04758 | 0.338871 |
| *AHL13-1* | Gh_A08G1421.1 | Ga08G1855.1 | 0.995804 | 1.01069 | 0.985274 | 0.809045 |
| *AHL13-2* | Gh_A12G1901.1 | Ga12G0709.1 | 0.976747 | 1.0691 | 0.913614 | 0.041726 |
| *AHL5-1* | Gh_A08G2384.1 | Ga08G2140.1 | 0.969015 | 1.08272 | 0.894981 | 0.019016 |
| *AHL5-2* | Gh_A12G2087.1 | Ga12G0508.1 | 1.00973 | 0.973768 | 1.03693 | 0.44097 |
| *AHL9-1* | Gh_A08G1169.1 | Ga08G1564.1 | 0.979564 | 1.05655 | 0.927138 | 0.132116 |
| *AHL9-2* | Gh_A11G3057.1 | Ga11G3138.1 | 0.981647 | 1.05193 | 0.933184 | 0.135726 |
| *AHL9-3* | Gh_A12G0982.1 | Ga12G1830.1 | 0.974612 | 1.07242 | 0.908798 | 0.038696 |
| *AHLx-1* | Gh_A01G1159.1 | Ga01G1578.1 | 1.00384 | 0.989272 | 1.01472 | 0.686804 |
| *AHLx-2* | Gh_A09G2445.1 | Ga09G1559.1 | 0.994179 | 1.01654 | 0.977999 | 0.642806 |
| *AHLx-3* | Gh_A04G0557.1 | Ga04G0938.1 | 0.952546 | 1.13479 | 0.8394 | 0.000162 |
| *AHLx-4* | Gh_A07G0971.1 | Ga07G1158.1 | 0.998191 | 1.00554 | 0.992695 | 0.933799 |
| *AHLx-5* | Gh_A05G3407.1 | Ga04G1890.1 | 0.984671 | 1.05735 | 0.931259 | 0.239266 |
