# Supplementary material for: Genome-wide identification and analyses of the AHL gene family in cotton (Gossypium)
Source: BMC Genomics. 2020 Jan 22;21:69. doi: 10.1186/s12864-019-6406-6 (PMC6977275; doi:10.1186/s12864-019-6406-6)
Supplement: Supplementary file 11 — Additional file 11. - The primers designed for qRT-PCR [file 12864_2019_6406_MOESM11_ESM.docx]

**Additional files 11**

**The primers designed for qRT-PCR**

| Gene name | primer | sequence |
| --- | --- | --- |
| *AHL20-2* | Forward | GCTCACCGTGTACTTAGCTGGTG |
|  | Reverse | GGTGAATCGTTTGCTCCGCCTTG |
| *AHL22-1* | Forward | CGACACGAGTGAAAGCAAAGAAG |
|  | Reverse | CATCAGAGCCGTTGGCAATCTC |
| *AHL16-1* | Forward | CAATTGTGACTTTACATGGTCGG |
|  | Reverse | GCTGCTGTTGTTGCAACTTCATCAG |
| *AHL9-1* | Forward | GGCATTATCAGGGTCTGCATCTTAC |
|  | Reverse | CTAAGGTTGATCCCACAGTAGT |
| *AHL7-1* | Forward | CACCAGTCGATGGTTTAACACCAGTC |
|  | Reverse | CCAATAGAAGTGCCGGCCAACTCAC |
| *AHL10* | Forward | GCAAGCTCTGAACATGAGCATGAG |
|  | Reverse | CTGAAGTTGGTGAAGAGGCTGATC |
| *Actin* | Forward | ATCCTCCGTCTTGACCTTG |
|  | Reverse | TGTCCGTCAGGCAACTCAT |
